# Supplementary material for: Lipolysis-derived fatty acids are needed for homeostatic control of sterol element-binding protein-1c driven hepatic lipogenesis
Source: Commun Biol. 2025 Apr 9;8:588. doi: 10.1038/s42003-025-08002-1 (PMC11982389; doi:10.1038/s42003-025-08002-1)
Supplement: Supplementary file 1 — Supplementary Information [file 42003_2025_8002_MOESM1_ESM.pdf]

A

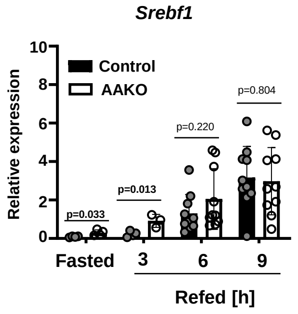

B

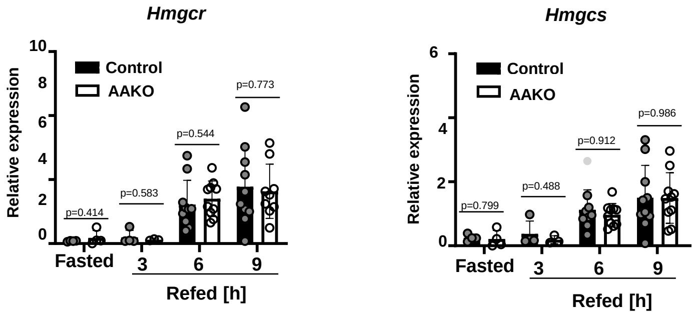

Supplementary Figure 1. AAKO qPCR Supplement Groups of Control and AAKO (adipose specific ATGL deficient) mice were fasted for 9 h overnight and subsequently either sacrificed (Fasted) or refed a HChD (high carbohydrate/low-fat diet) and sacrificed at the time points indicated (Refed). **(A)** Liver *Srebf1* (mRNA) and **(B)** SREBP-2 target genes; *Hmgcr* and *Hmgcs* (mRNA) levels were measured by qPCR (quantitative real-time Polymerase Chain Reaction). n=3-10/group. Outliers are shown as light grey dots in the graph. Unpaired T-tests were used to compute significance levels.

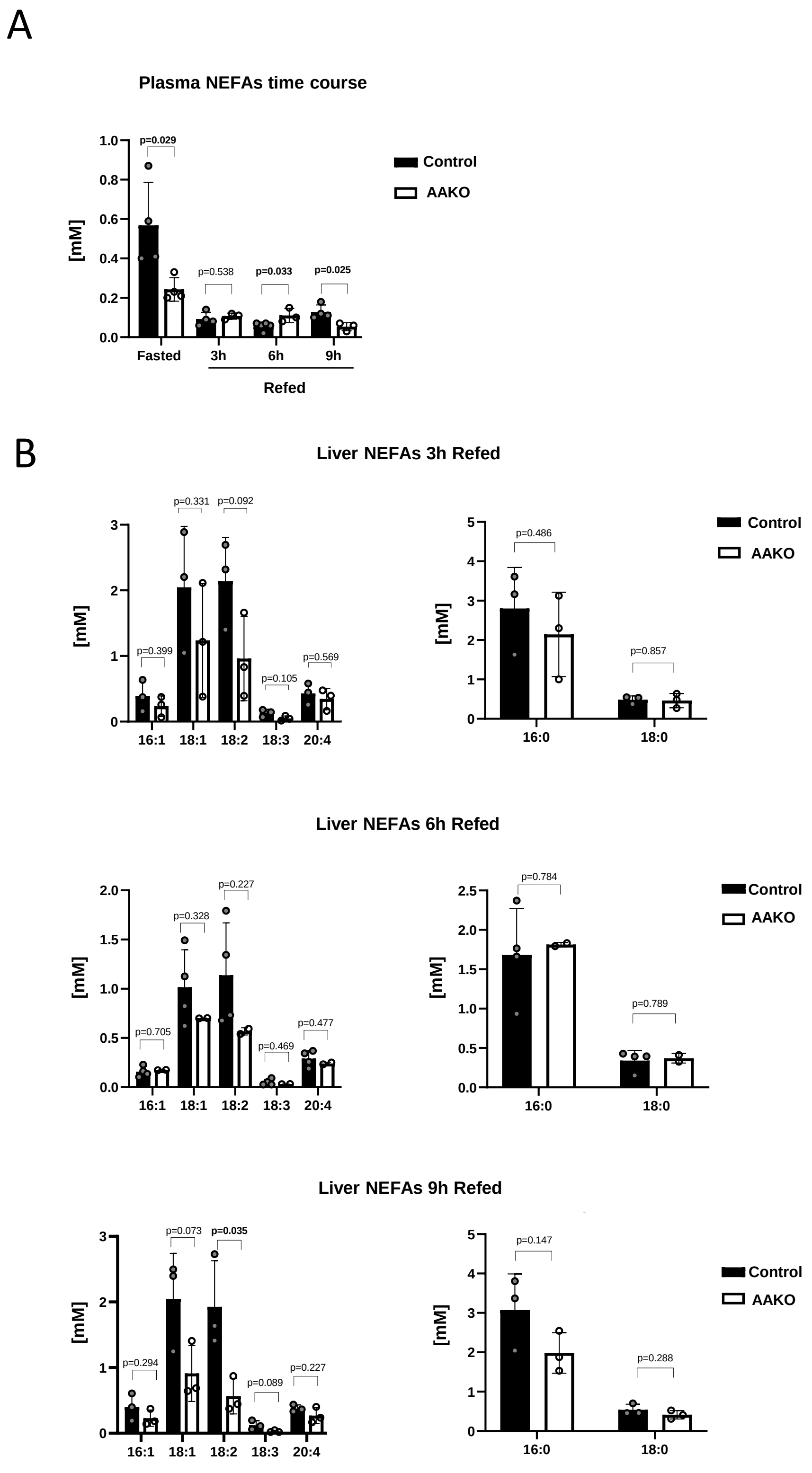

Supplementary Figure 2. AAKO Liver free fatty acids. Control mice and AAKO (adipose specific ATGL deficient) mice were fasted for 9 h overnight and subsequently either sacrificed (Fasted) or refed a HChD (high carbohydrate/low-fat diet) and sacrificed at the time points indicated (Refed). Blood was drawn and livers were resected. **(A)** Plasma NEFA (non-esterified fatty acids) levels and **(B)** liver NEFA levels, were measured using GC/FID (Gas Chromatography-Flame Ionization Detection). n=3-4/group. Unpaired T-tests were used to compute significance levels.

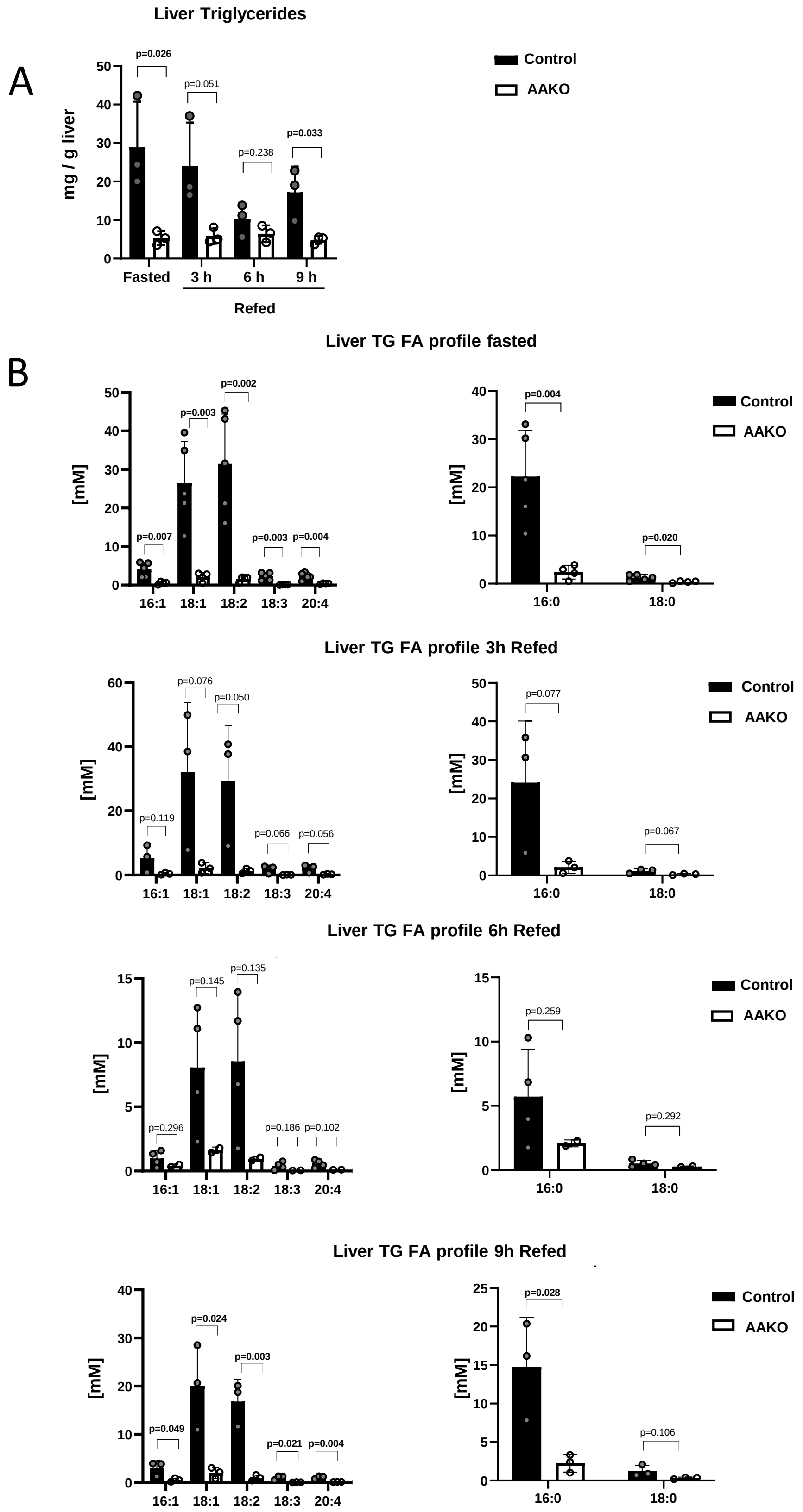

Supplementary Figure 3. AAKO Liver Triglycerides (TG). Control mice and AAKO (adipose specific ATGL deficient) mice were fasted for 9 h overnight and subsequently, either sacrificed (Fasted) or refed a HChD (high carbohydrate/low-fat diet) and sacrificed at the time points indicated (Refed). Livers were resected. **(A)** Total liver TG content, and **(B)** liver TG fatty acid (FA) profiles (FAs incorporated in TGs) from control and AAKO mouse livers, were measured using GC/FID (Gas Chromatography-Flame Ionization Detection). n=3-5/group. Unpaired T-tests were used to compute significance levels.

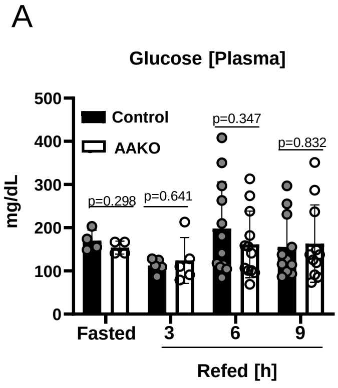

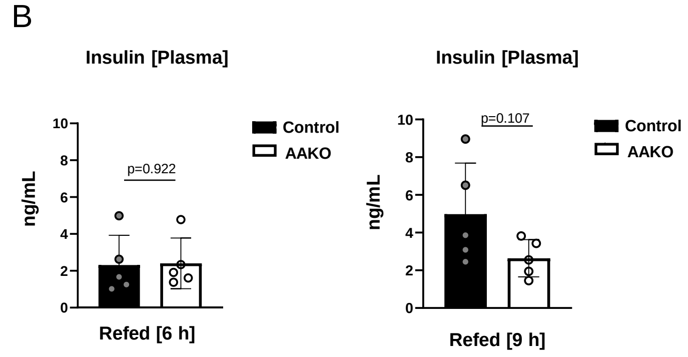

Supplementary Figure 4. AAKO Plasma glucose and insulin. Groups of Control and AAKO (adipose specific ATGL deficient) mice were fasted for 9 h overnight and subsequently, either sacrificed (Fasted) or refed a HChD (high carbohydrate/low fat diet) and sacrificed at the time points indicated (Refed). Blood was withdrawn and plasma was obtained. **(A)** Plasma glucose concentration was measured in frozen samples using a glucometer. **(B)** Plasma insulin levels were determined using a mouse insulin ELISA. n=4-12 / group. Unpaired T-tests were used to compute significance levels.

A

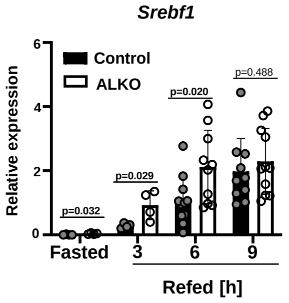

B

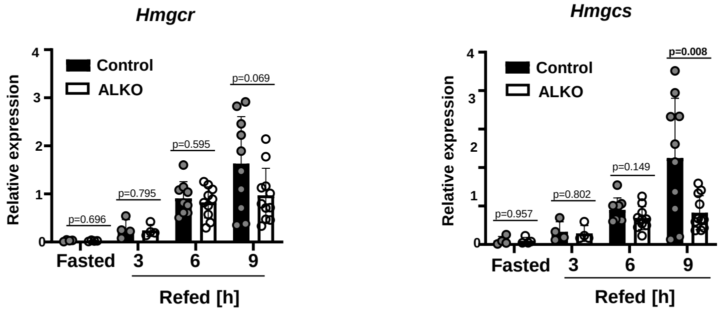

Supplementary Figure 5. ALKO qPCR Supplement. Control and ALKO (liver specific ATGL deficient) mice were fasted for 9 h overnight and subsequently, either sacrificed (Fasted) or refed a HChD (high carbohydrate/low-fat diet) and sacrificed at the time points indicated (Refed). Livers were resected. **(A)** qPCR, gene expression of *Srebf1* and the **(B)** SREBP-2 target genes *Hmgcr* and *Hmgcs.* n=4-10 / group. Unpaired T-tests were used to compute significance levels.

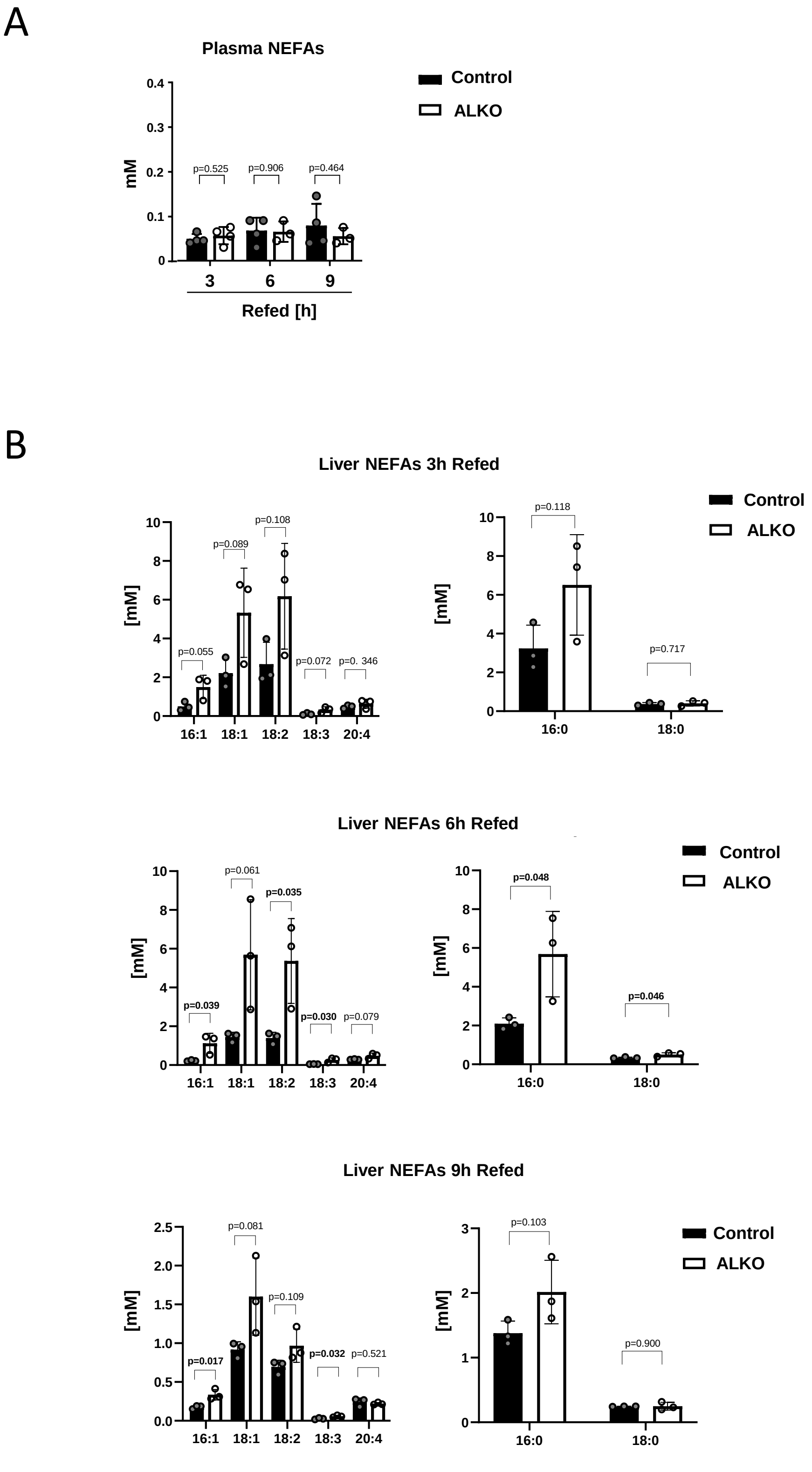

Supplementary Figure 6. ALKO Liver free fatty acids. Control and ALKO (liver specific ATGL deficient) mice were fasted for 9 h overnight, subsequently refed a HChD (high carbohydrate/low-fat diet) and sacrificed at the time points indicated (Refed). Blood was drawn and livers were resected. **(A)** Plasma NEFA (non-esterified fatty acids) levels and **(B)** Liver NEFA levels were measured using GC/FID (Gas Chromatography-Flame Ionization Detection). n=3-4/group. Unpaired T-tests were used to compute significance levels.

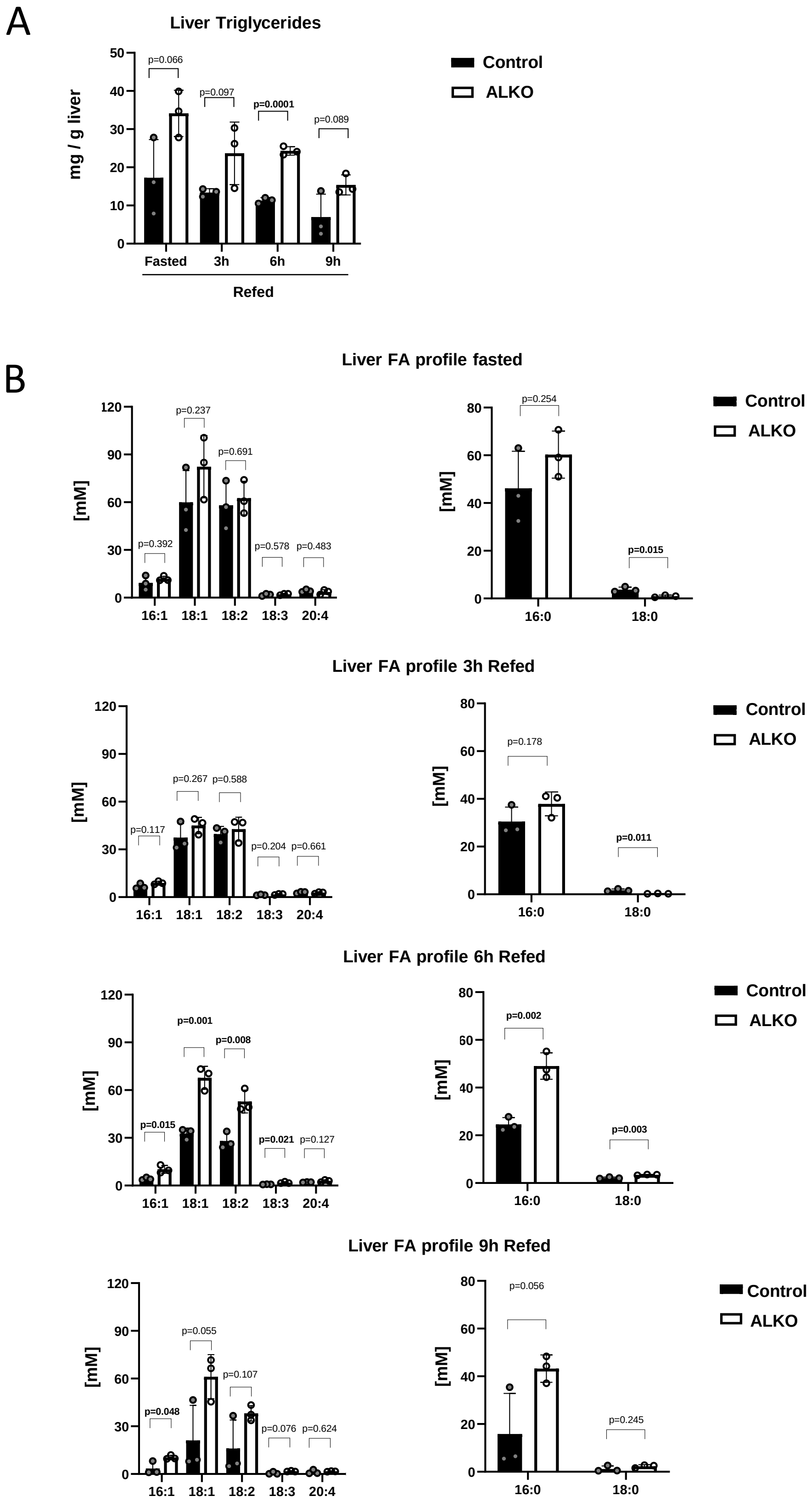

Supplementary Figure 7. ALKO Liver Triglycerides. Control and ALKO (liver specific ATGL deficient) mice were fasted for 9 h overnight, subsequently either sacrificed (Fasted) or refed a HChD (high carbohydrate/low-fat diet), and sacrificed at the time points indicated (Refed). Livers were resected. **(A)** Total triglyceride content, and **(B)** liver TG fatty acid (FA) profiles (FAs incorporated in TGs) from control and AAKO mouse livers, were measured using GC/FID (Gas Chromatography-Flame Ionization Detection). n=3-4/group. Unpaired T-tests were used to compute significance levels.

A

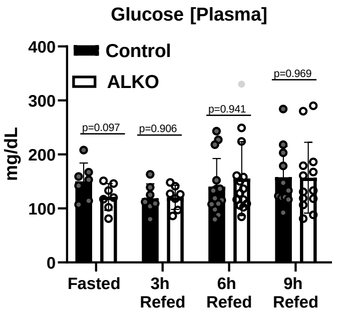

B

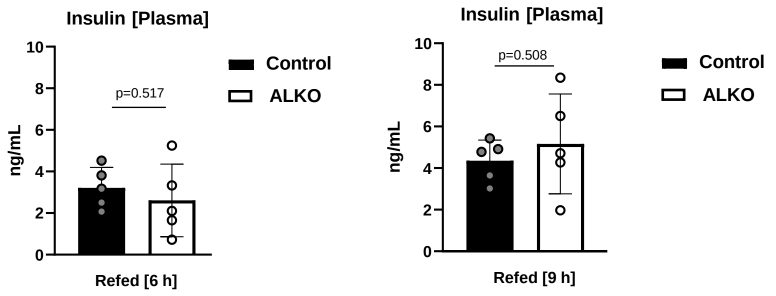

Supplementary Figure 8. ALKO Plasma glucose and insulin. Control and ALKO (liver specific ATGL deficient) mice were fasted for 9 h overnight and subsequently, either sacrificed (Fasted) or refed a HChD (high carbohydrate diet), and sacrificed at the time-points indicated (Refed). Blood was drawn and plasma was obtained. (**A**) Glucose concentration was measured from frozen plasma samples using a glucometer. (**B**) Plasma insulin levels were determined using a mouse insulin ELISA. n=5-12 / group. Outliers are shown as light grey dots. Unpaired T-tests were used to compute significance levels.

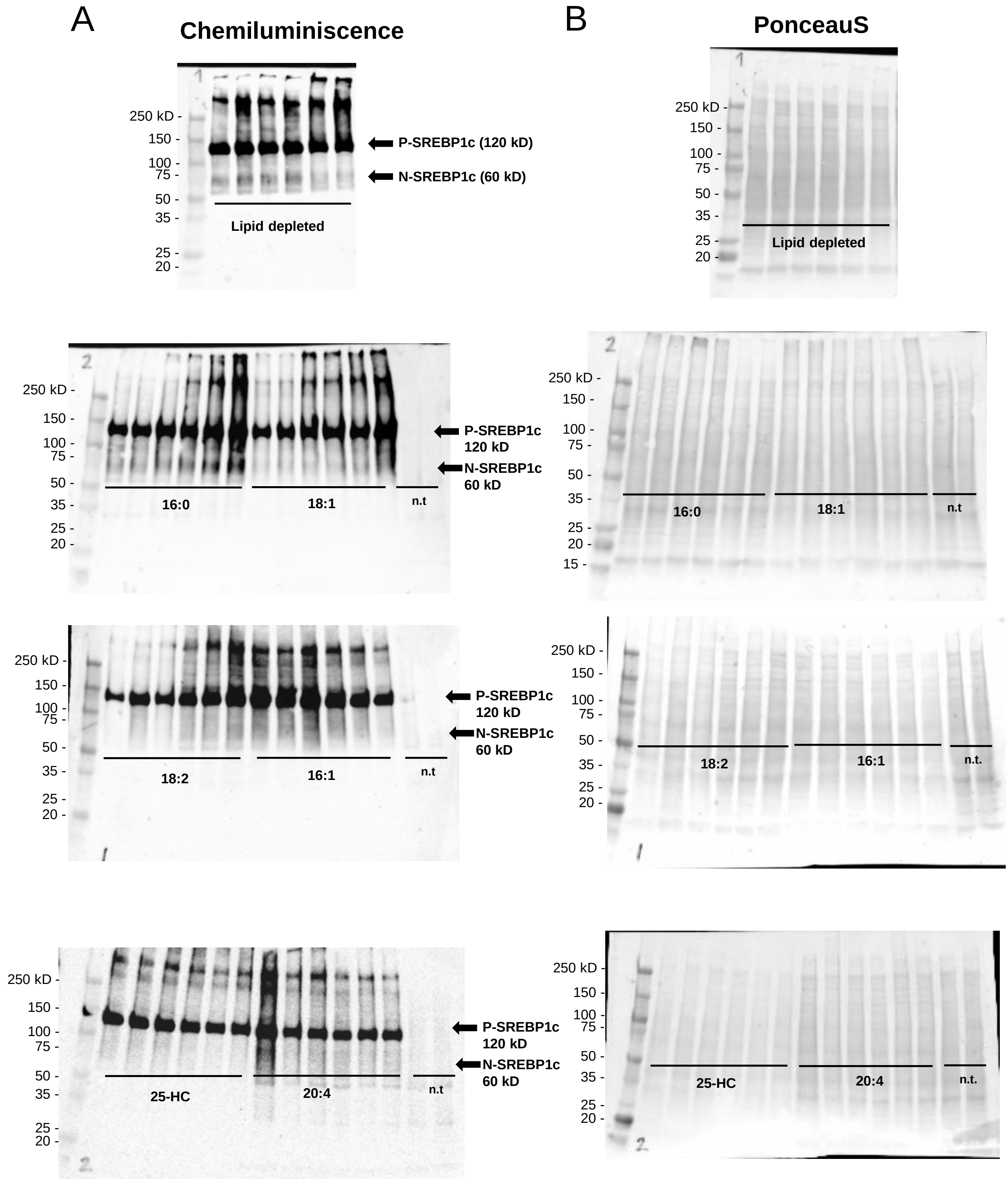

Supplementary Figure 9. The pCDNA3.1-Flag-SREBP-1c vector construct contains a triple Flag-tag in front of the human *SREBF1* cDNA (Flag-SREBP-1c) and was transiently transfected into HepG2 cells (Figure 3 A). 48 hours after transfection, HepG2 cells were incubated in lipid depleted medium (5% LPDS) or 5% LPDS plus the addition of 100 µM saturated fatty acids (16:0) or unsaturated fatty acids (18:1, 18:2, 16:1 or 20:4, respectively), or the SREBP-1c cleavage suppressor 25-hydroxycholesterol (25-HC), and incubated for 16 h. Non-transfected HepG2 cells were used as Flag-negative control (n.t). 2 h before harvest, the protease inhibitor ALLN (N-acetyl-leucinyl-leucinyl-norleucinal) was added. **(A)** Whole cell extracts were subjected to Western Blot (WB). P-SREBP-1c (120 kD) and N-SREBP-1c (60 kD) were detected using anti-Flag antibody on the same membrane. **(B)** Ponceau-S-stained membranes are shown as loading controls.

A

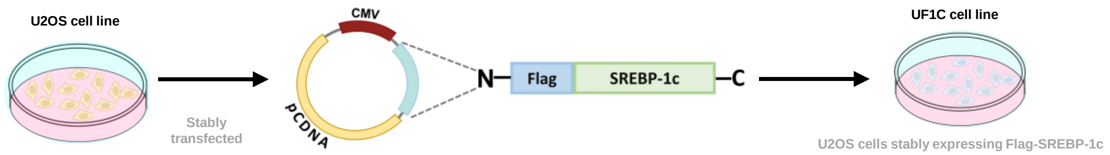

B

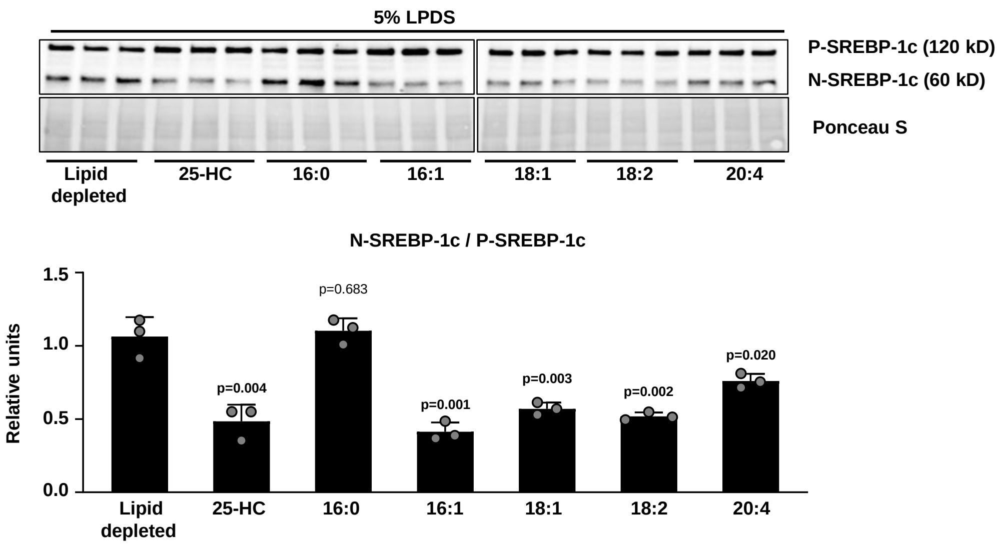

Supplementary Figure 10. Flag-SREBP1c cleavage reporter vector validation in U2OS cells. (A) We constructed a pCDNA3.1-Flag-SREBP-1c vector and used it to stably transfect and clonally select U2OS cells, yielding the stably Flag-SREBP-1c expressing UF1c cell line. **(B)** UF1c cells were incubated in lipid depleted medium (5% LPDS) or 5% LPDS plus the addition of 2.5 µM 25-HC, or 100 µM FAs (as indicated) for 16 h. 2 h before harvest, the protease inhibitor ALLN (N-acetyl-leucinyl-leucinyl-norleucinal) was added. Whole cell extracts were subjected to Western Blot. P- and N-SREBP-1c were detected using anti-Flag antibody. Band intensities were measured using ImageJ, NIH. Relative levels of proteolytically cleaved N-SREBP-1c were calculated as the relative fraction of N-SREBP-1c / P-SREBP-1c signal intensities, and are presented in the dot plot, n=3/group.

A

B

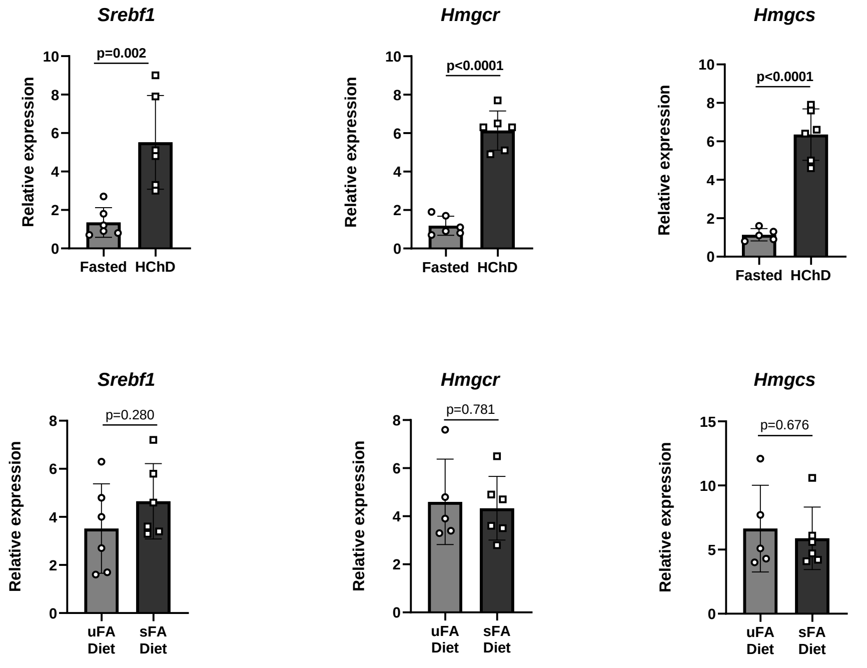

Supplementary Figure 11. Wild type mice were injected intravenously with $2x10^9$ PFU AdFlag-SREBP-1c reporter vector. Four days later the mice were (**A**) either fasted overnight or fasted and refed a high carbohydrate diet (HChD) overnight, or (**B**) fed for 3 consecutive days with a chow diet enriched with palm oil (saturated fatty acid diet, sFA) or flaxseed oil (unsaturated fatty acid diet, uFA). Liver *Srebf1* (mRNA) and SREBP-2 target genes *Hmgcr* and *Hmgcs* mRNA levels were measured by qPCR (quantitative real-time Polymerase Chain Reaction). n=6 mice/group. Unpaired T-tests were used to compute significance levels.

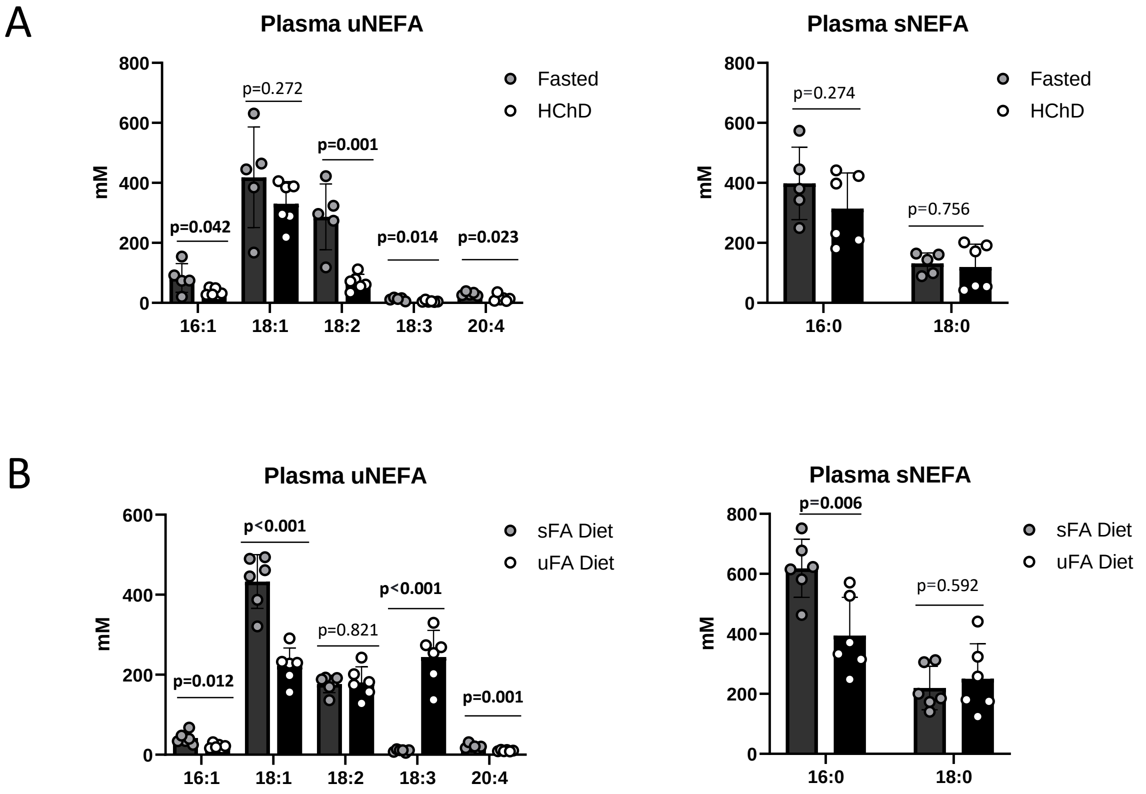

Supplementary Figure 12. Plasma Non-esterified fatty acid (NEFA) profile. Wild type mice were injected intravenously with 2x10^9 PFU AdFlag-SREBP-1c reporter vector. Four days later the mice were (**A**) either fasted overnight or fasted and refed a high carbohydrate diet (HChD) overnight, or (**B**) fed for 3 consecutive days with a chow diet enriched with palm oil (saturated fatty acid diet, sFA) or flaxseed oil (unsaturated fatty acid diet, uFA). Blood was drawn and plasma was obtained. Plasma unsaturated NEFA (uNEFA) or saturated NEFA (sNEFA) levels were measured in **(A)** fasted or HChD groups, or **(B)** uFA or sFA diet groups using GC/FID (Gas Chromatography-Flame Ionization Detection), n=5-6/group. Unpaired T-tests were used to compute significance levels.

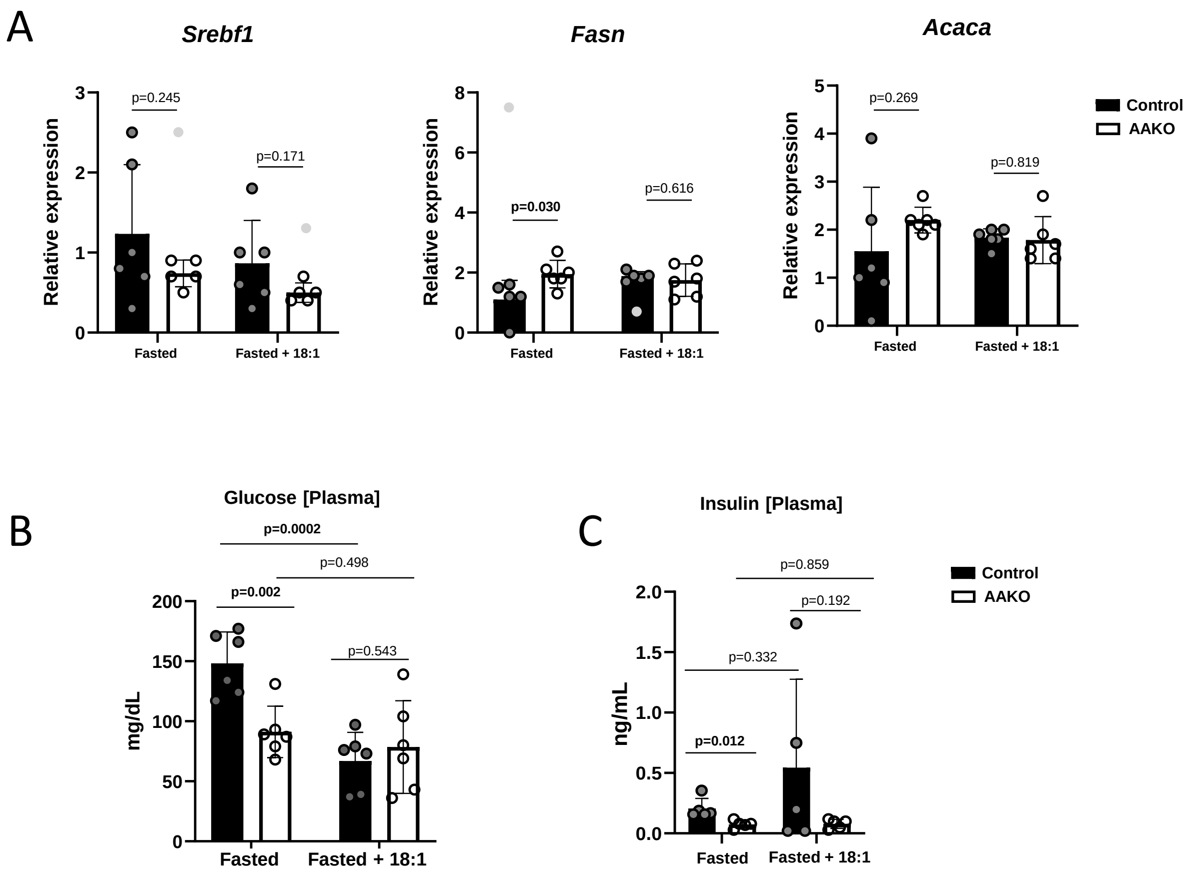

Supplementary figure 13. Plasma insulin and glucose levels. Control and AAKO (adipose specific ATGL deficient) mice were fasted for 12 h overnight and subsequently, either sacrificed (Fasted) or fasted overnight and injected with bovine serum albumin complexed oleic acid (18:1). **(A)** Liver *Srebf1*, *Acaca* and *Fasn* mRNA levels were determined by qPCR (quantitative real-time Polymerase Chain Reaction). Outliers are shown as light grey dots. n=6/ group. Unpaired T-tests were used to compute significance levels. **(B)** Plasma glucose levels were determined by glucometer from frozen plasma samples n=6 / group **(C)** Plasma insulin levels were determined by mouse insulin ELISA. n=5 / group. Unpaired T-tests were used to compute significance levels.

Figure 1.

Figure 1A. SREBP-1c labeled western blot images Fasted.

Ponceau S Membrane Extracts (MM) Fasted

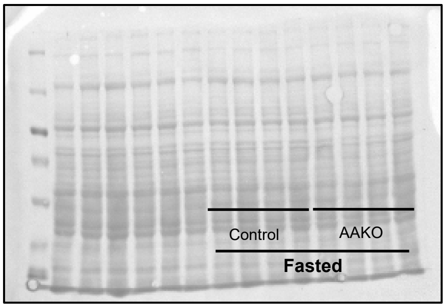

Chemiluminescence

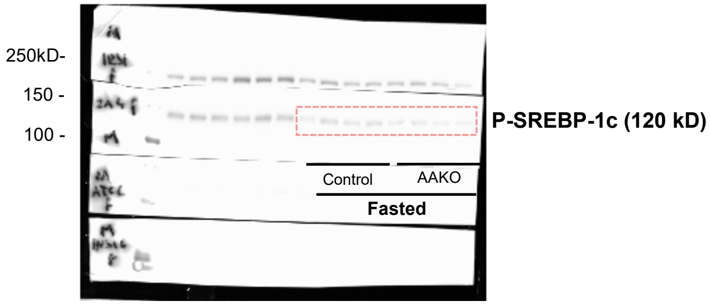

Ponceau S Nuclear Extracts (NEX) Fasted

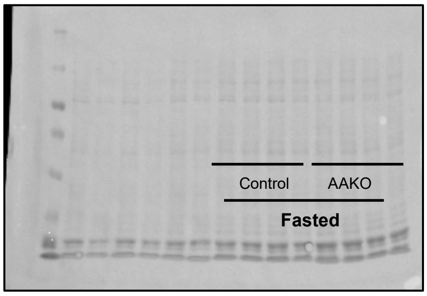

Chemiluminescence

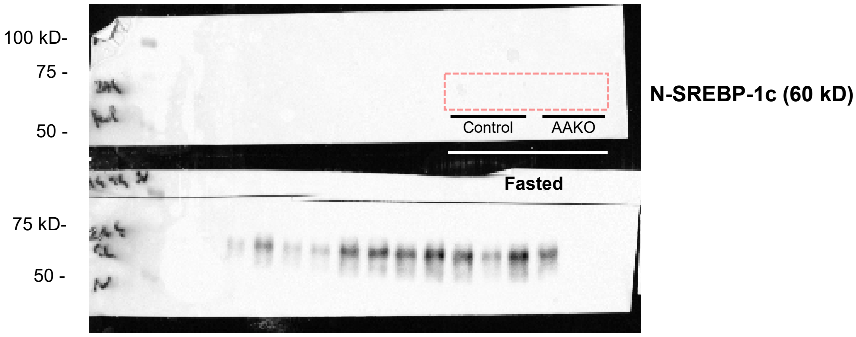

Supplementary Figure 14. Uncropped and unedited Western Blot images Figure 1A.

Figure 1A. SREBP-1c labeled western blot images 3h and 6h Refed .

Ponceau S Membrane Extracts (MM)
3h and 6h Refed

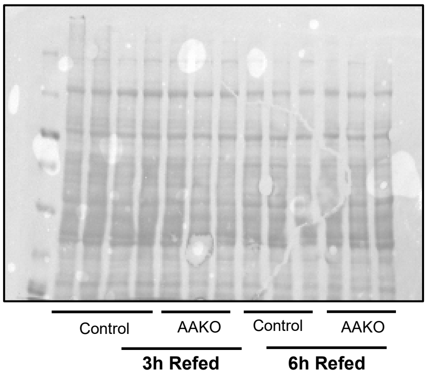

Chemiluminescence

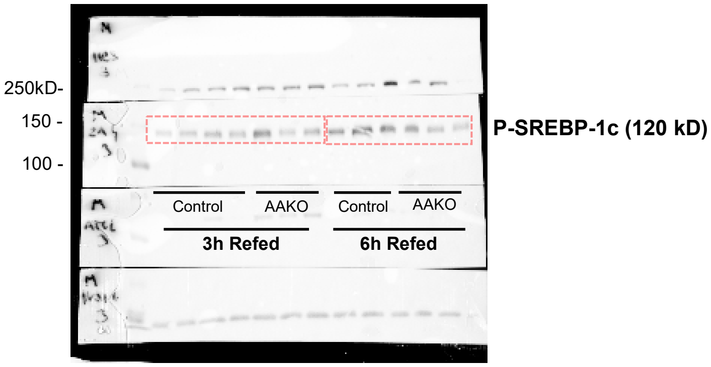

Ponceau S Nuclear Extracts (NEX)
3h and 6h Refed

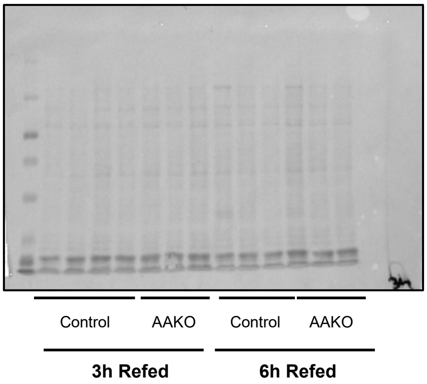

Chemiluminescence

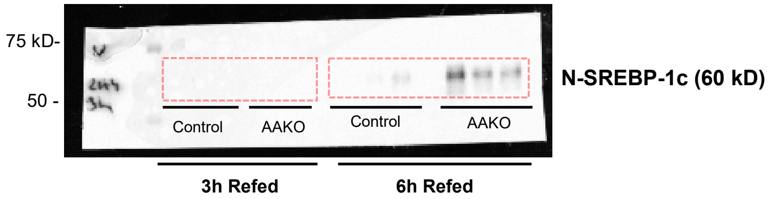

Supplementary Figure 14. Uncropped and unedited Western Blot images Figure 1A.

Figure 1A. SREBP-1c labelled western blot images 6h Refed .

Ponceau S Membrane Extracts (MM)
6h Refed

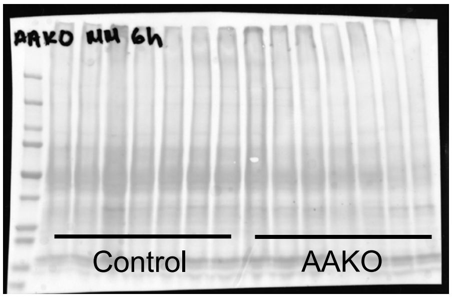

Chemiluminescence

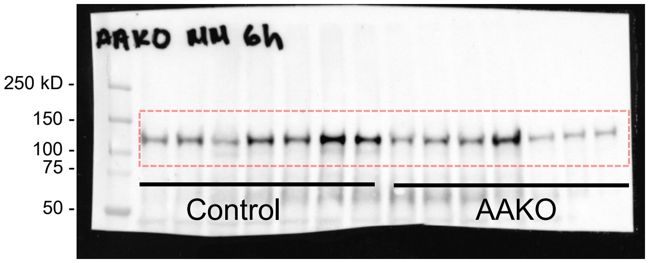

P-SREBP1c
(120 kD)

Ponceau S Nuclear Extracts (NEX)
6h Refed

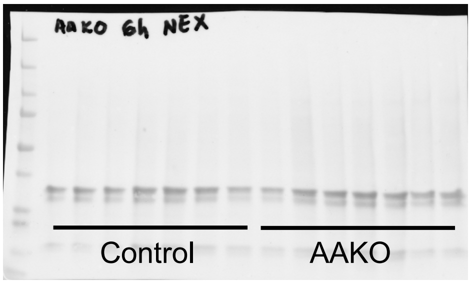

Chemiluminescence

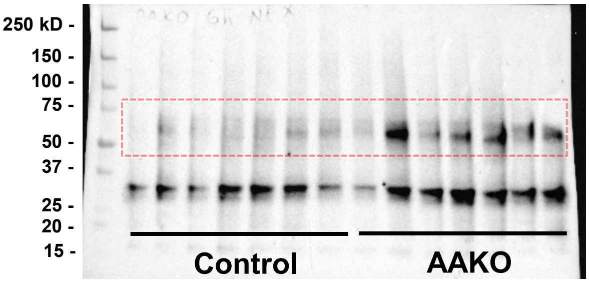

N-SREBP1c
(60 kD)

Supplementary Figure 14. Uncropped and unedited Western Blot images Figure 1A.

Figure 1A. SREBP-1c. labeled western blot images 9h Refed .

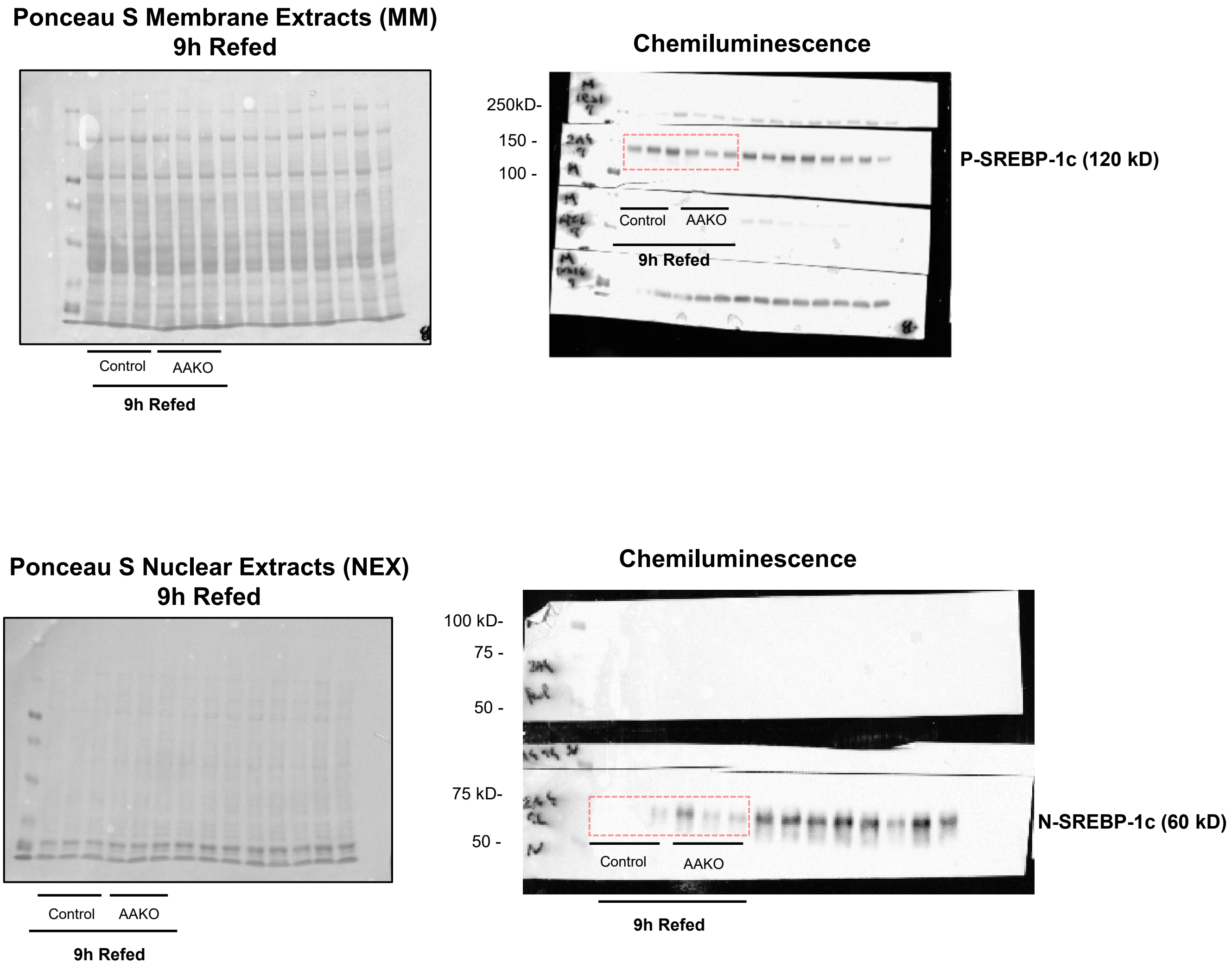

Supplementary Figure 14. Uncropped and unedited Western Blot images Figure 1A.

Figure 1A. SREBP-1c labeled western blot images 9h Refed.

Ponceau S Membrane Extracts (MM)
9h Refed

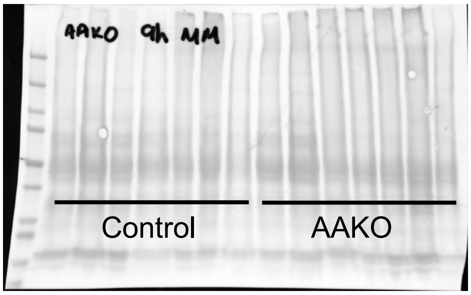

Chemiluminescence

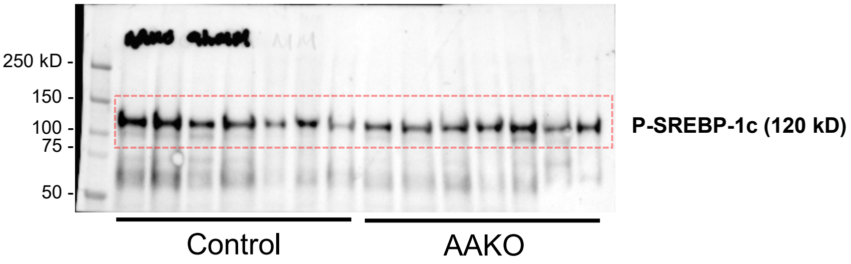

Ponceau S Nuclear Extracts (NEX)
9h Refed

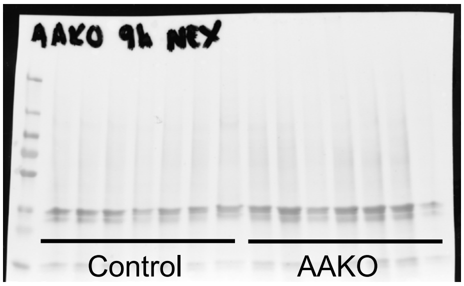

Chemiluminescence

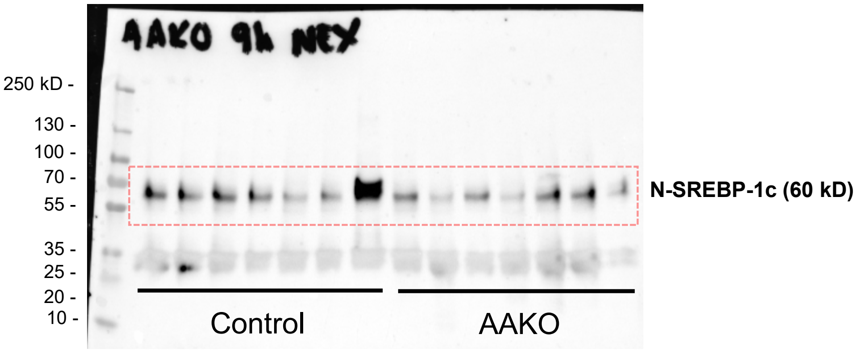

Supplementary Figure 14. Uncropped and unedited Western Blot images Figure 1A.

Figure 1G. Insulin signaling western blots 6h Refed

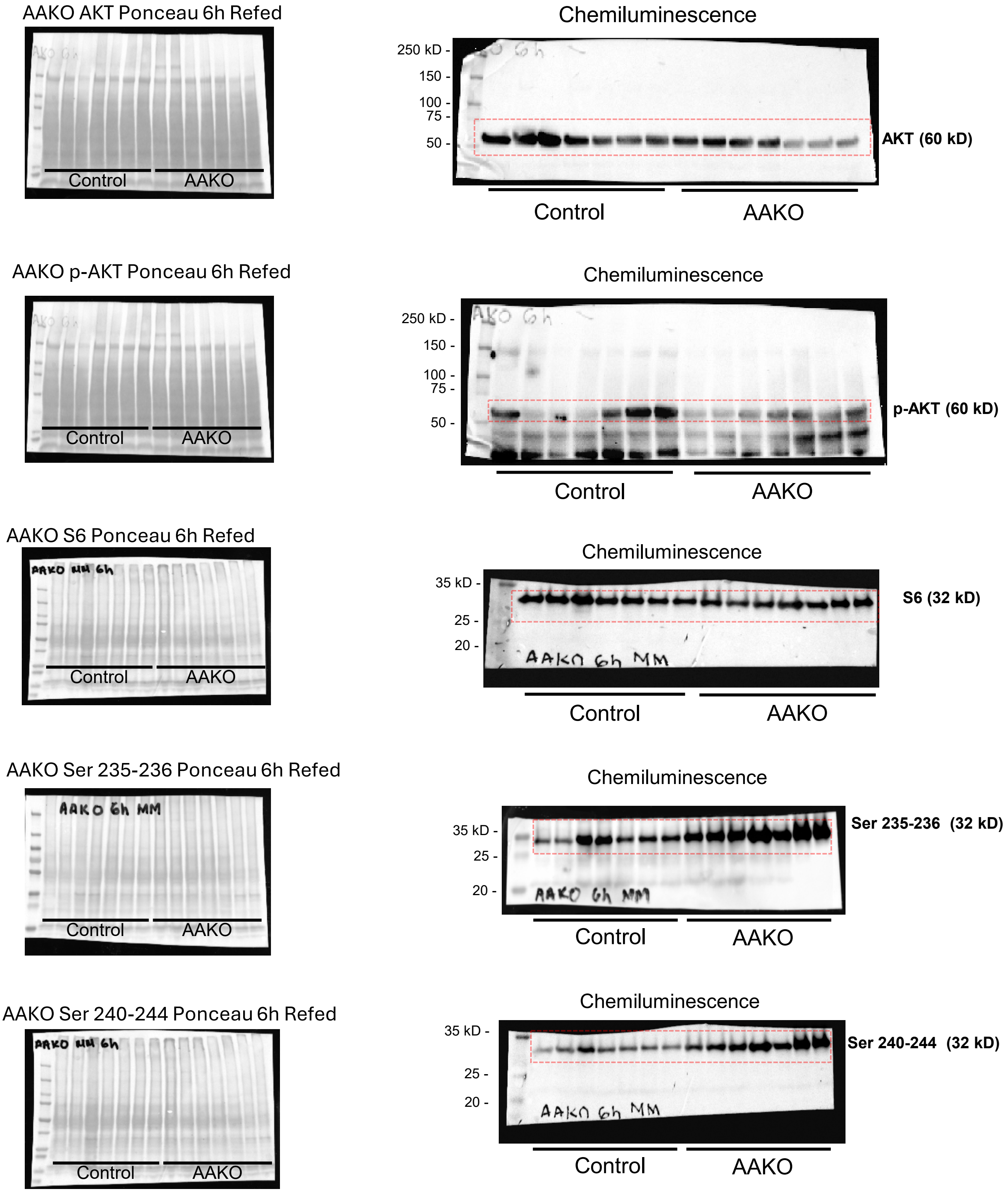

Supplementary Figure 14. Uncropped and unedited Western Blot images Figure 1G.

Figure 1G. Insulin signaling western blots 9h Refed

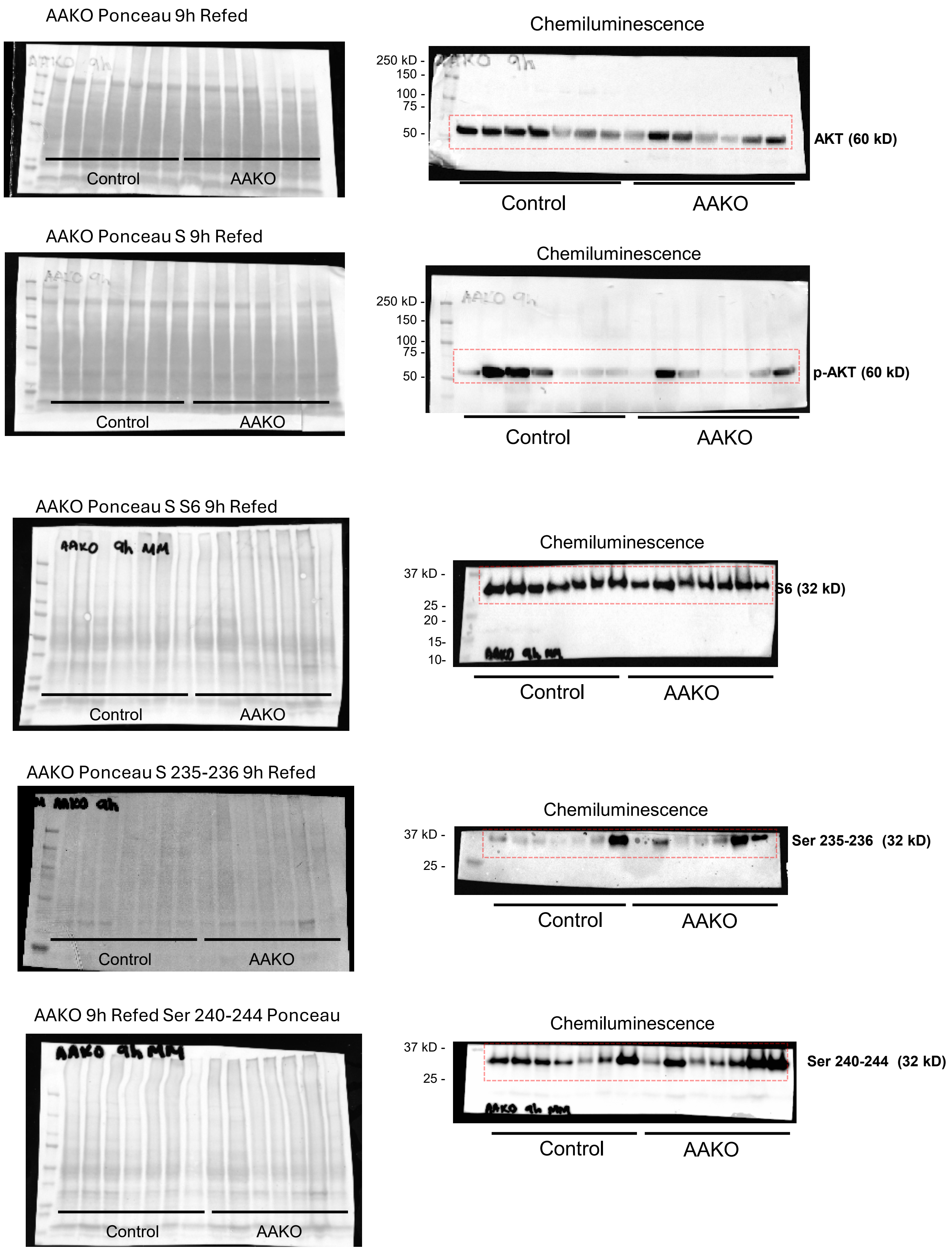

Supplementary Figure 14. Uncropped and unedited Western Blot images Figure 1G.

Figure 2.

Figure 2A. SREBP-1c labeled western blot images Fasted

Ponceau S Membrane Extracts (MM)
Fasted

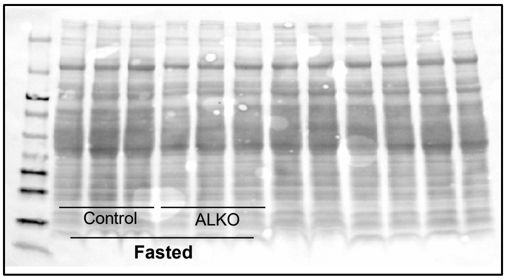

Chemiluminescence

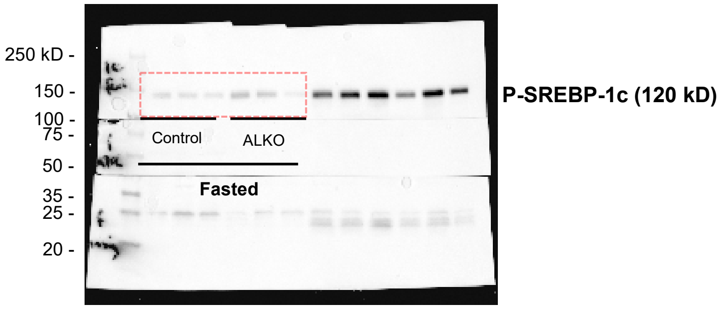

Ponceau S Nuclear Extracts (NEX)
Fasted

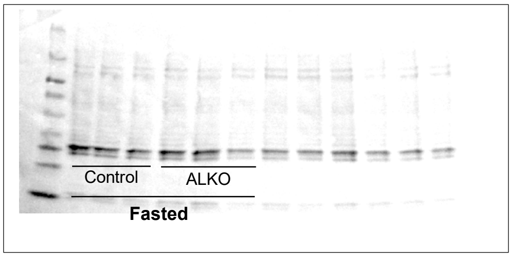

Chemiluminescence

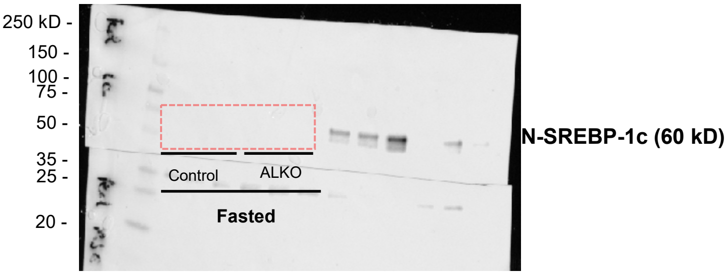

Supplementary Figure 14. Uncropped and unedited Western Blot images Figure 2A.

Figure 2A. SREBP-1c labeled western blot images 3h and 6h Refed

Ponceau S Membrane Extracts (MM)
3h and 6h Refed

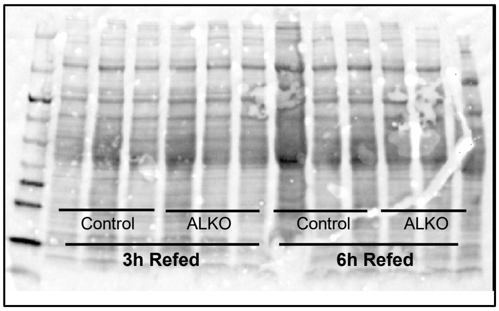

Chemiluminescence

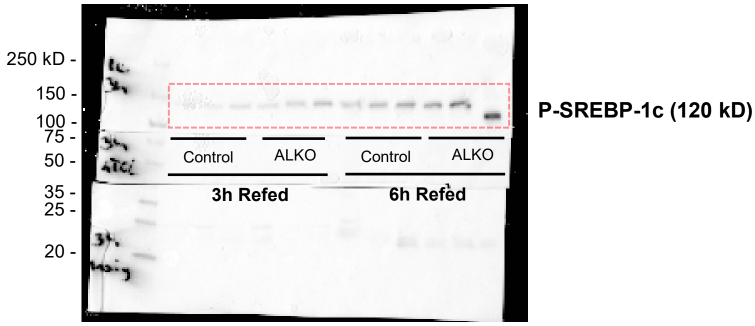

Ponceau S Nuclear Extracts (NEX)
3h and 6h Refed

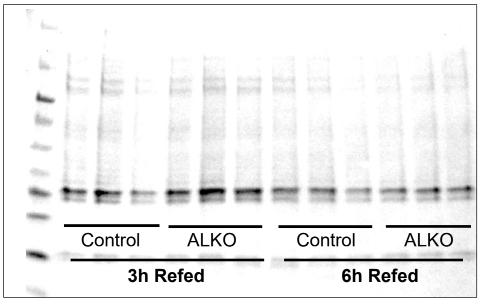

Chemiluminescence

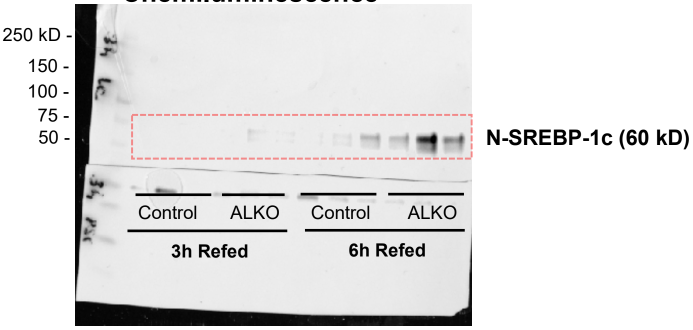

Supplementary Figure 14. Uncropped and unedited Western Blot images Figure 2A.

Figure 2A. SREBP-1c Labeled western blot images 6h Refed

Ponceau S Membrane Extracts (MM)
6h Refed

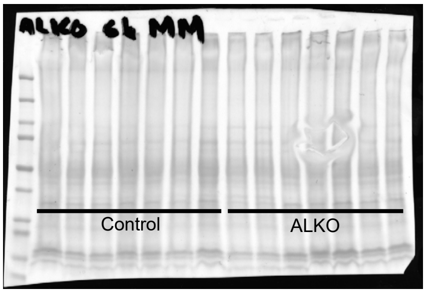

Chemiluminescence

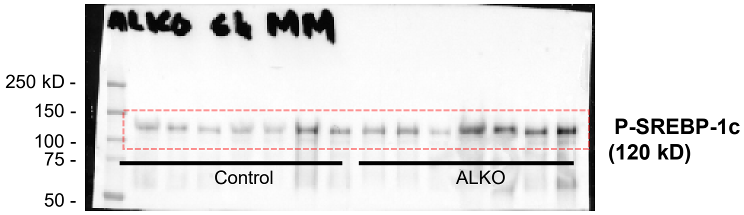

Ponceau S Nuclear Extracts (NEX)
6h Refed

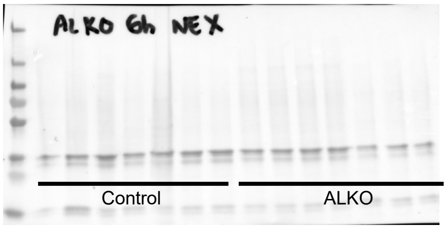

Chemiluminescence

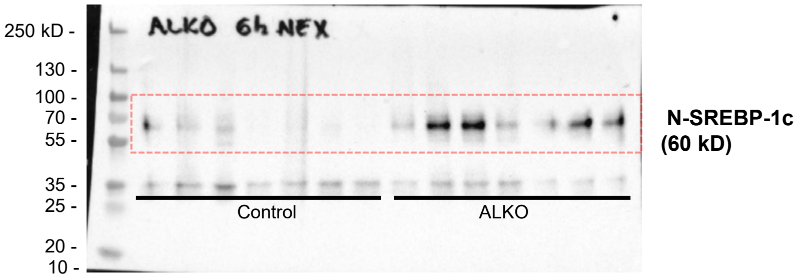

Supplementary Figure 14. Uncropped and unedited Western Blot images Figure 2A.

Figure 2A. SREBP-1c Labeled western blot images 9h Refed

Ponceau S Membrane Extracts (MM) 9h Refed

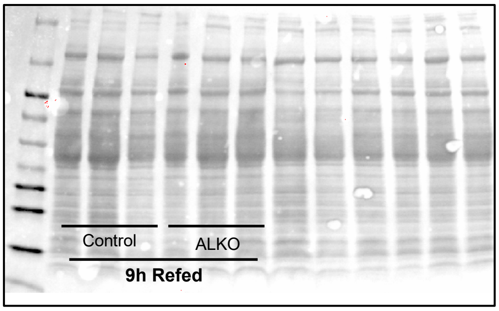

Chemiluminescence

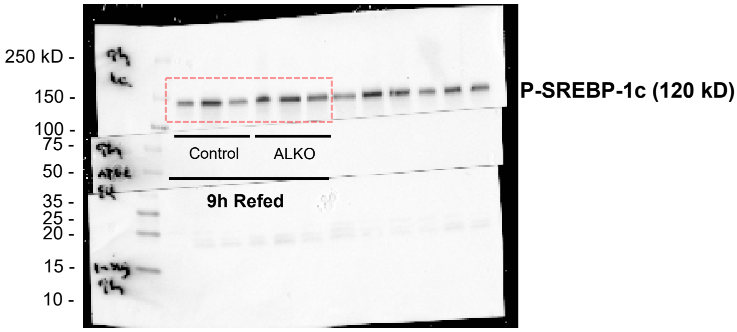

Ponceau S Nuclear Extracts (NEX) 9h Refed

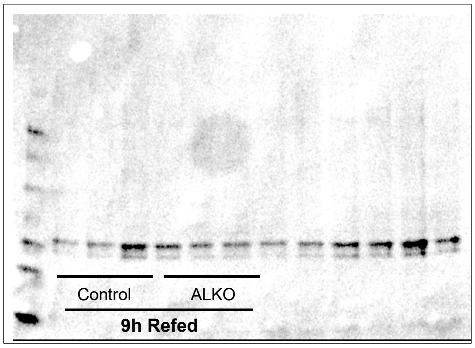

Chemiluminescence

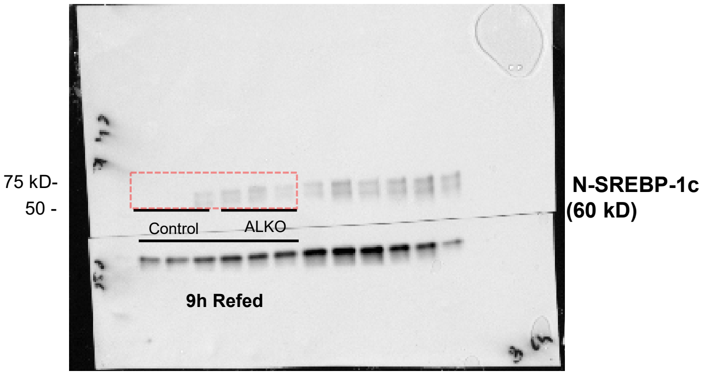

Supplementary Figure 14. Uncropped and unedited Western Blot images Figure 2A.

Figure 2A. SREBP-1c Labeled western blot images 9h Refed

Ponceau S Membrane Extracts (MM)
9h Refed

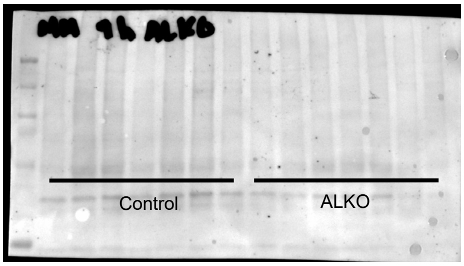

Chemiluminescence

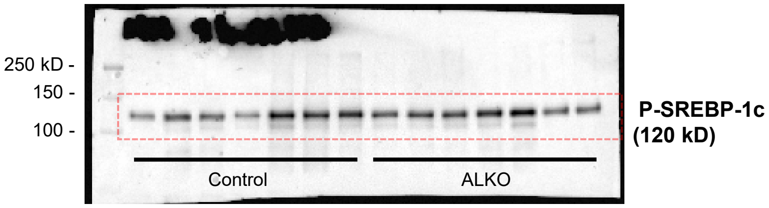

Ponceau S Nuclear Extracts (NEX)
9h Refed

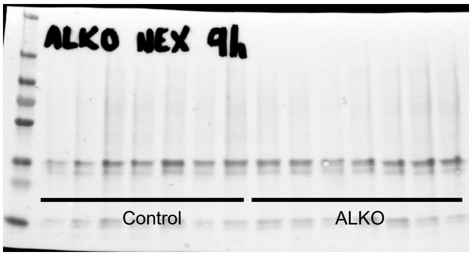

Chemiluminescence

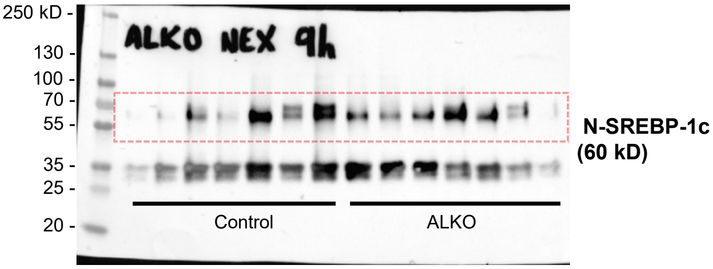

Supplementary Figure 14. Uncropped and unedited Western Blot images Figure 2A.

Figure 2G. Insulin signaling western blots 6h Refed

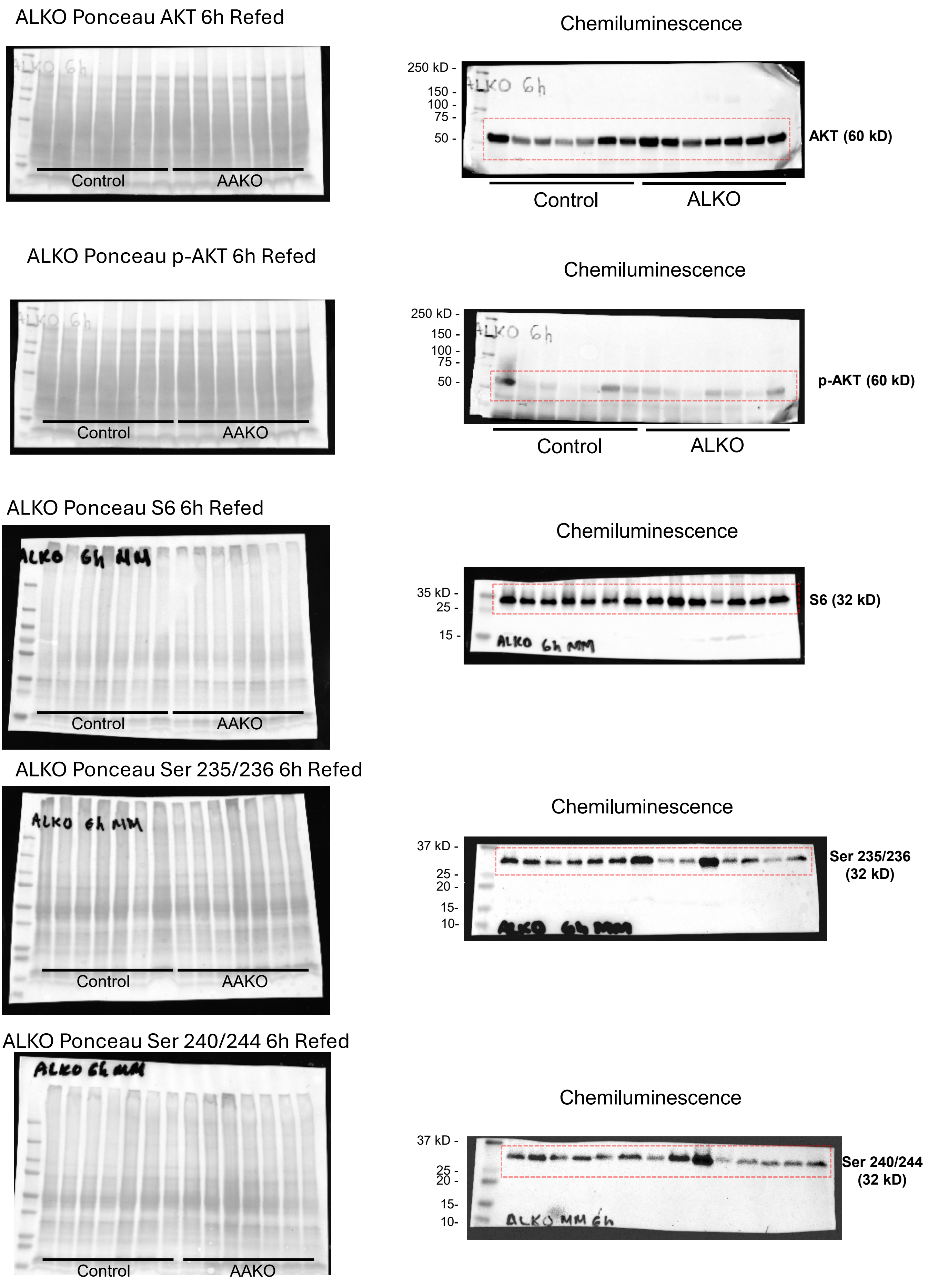

Supplementary Figure 14. Uncropped and unedited Western Blot images Figure 2G.

Figure 2G. Insulin signaling western blots 9h Refed

ALKO Ponceau AKT 9h Refed

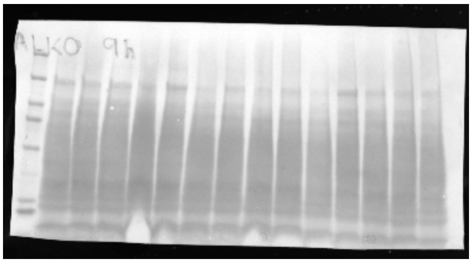

Chemiluminescence

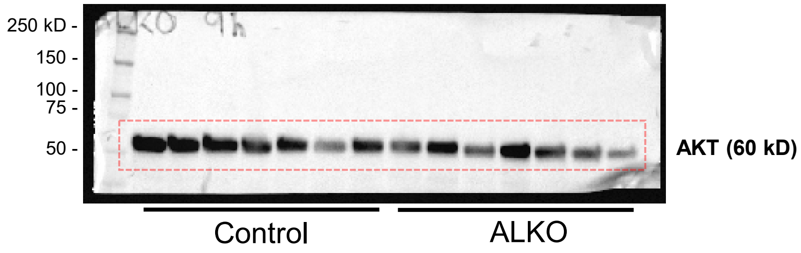

ALKO Ponceau p-AKT 9h Refed

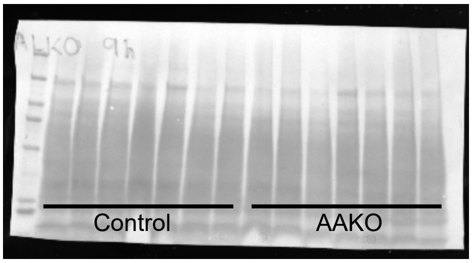

Chemiluminescence

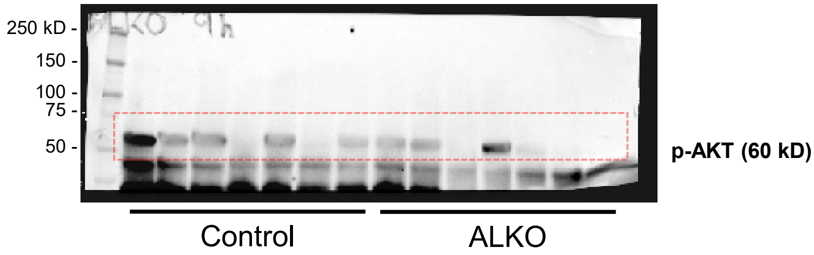

ALKO Ponceau S6 9h Refed

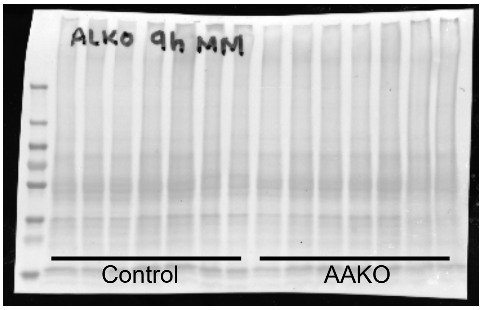

Chemiluminescence

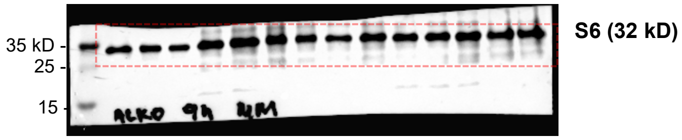

ALKO Ponceau P-S6 235/236 9h Refed

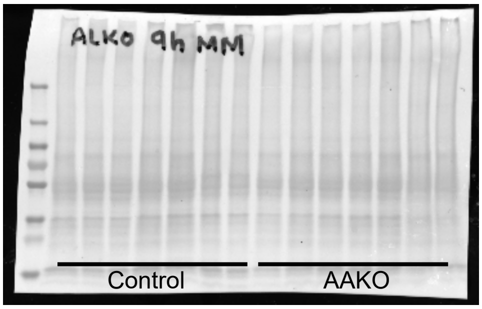

Chemiluminescence

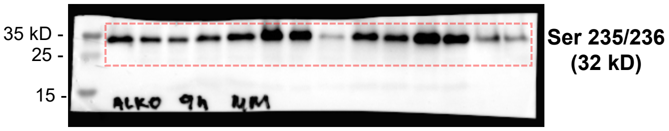

ALKO Ponceau P-S6 240/244 9h Refed

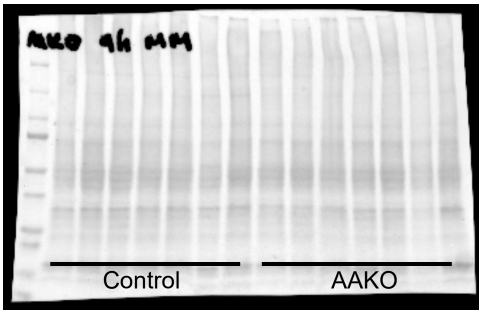

Chemiluminescence

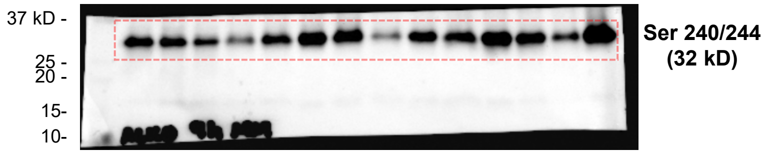

Supplementary Figure 14. Uncropped and unedited Western Blot images Figure 2G.

Figure 3.

Figure 3B. Flag-SREBP-1c labeled western blot images

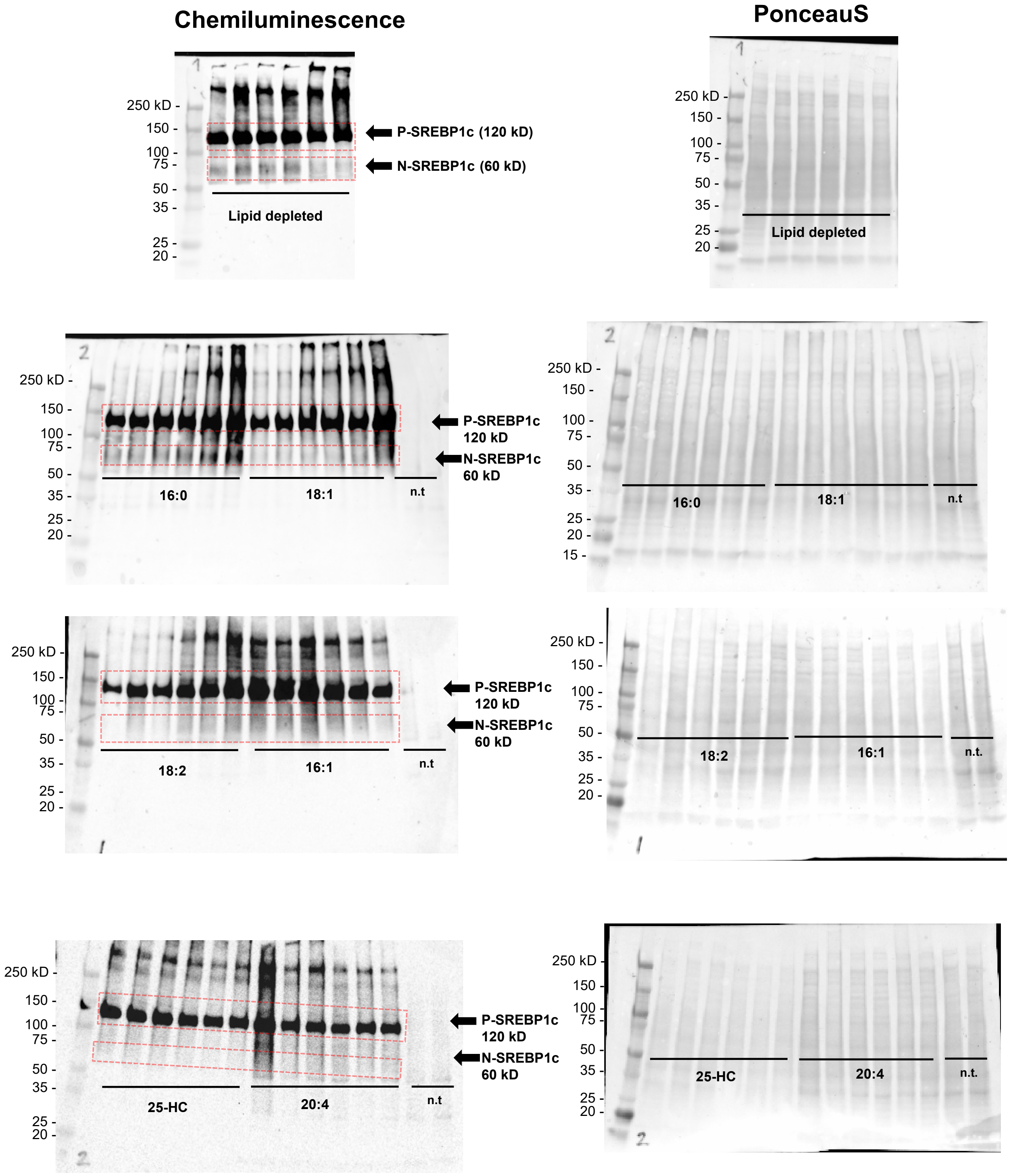

Supplementary Figure 14. Uncropped and unedited Western Blot images Figure 3B.

Figure 4.

Figure 4B. Flag-SREBP-1c labeled western blot images Fasted vs. High Carbohydrate Diet

Ponceau S Membrane Extract (MM)

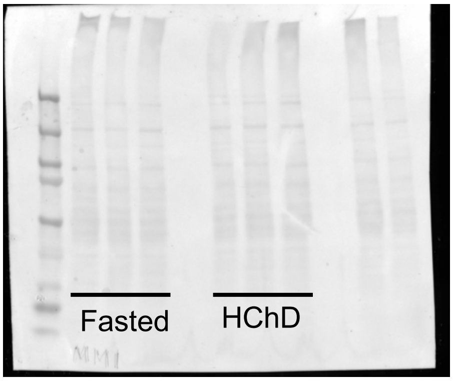

Chemiluminescence

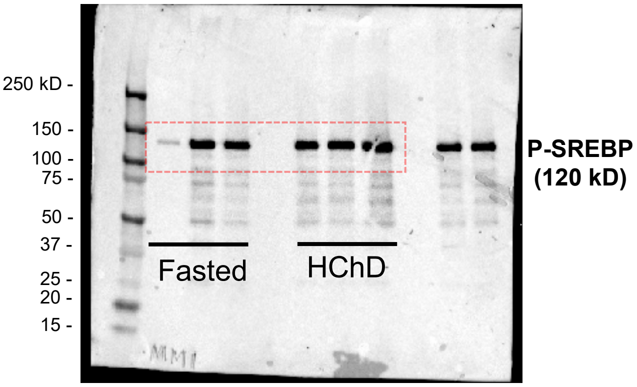

Ponceau S Nuclear Extract (NEX)

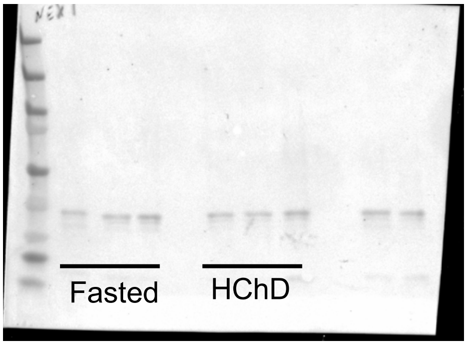

Chemiluminescence

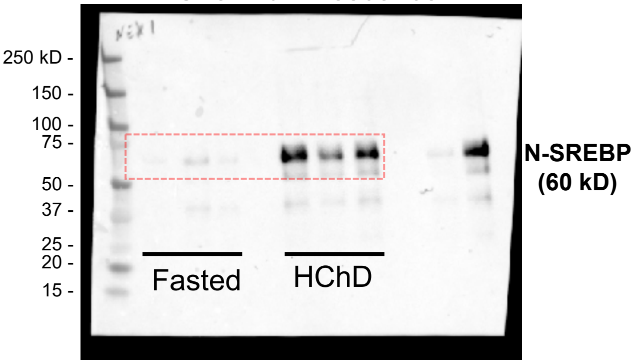

Ponceau S Membrane Extract (MM)

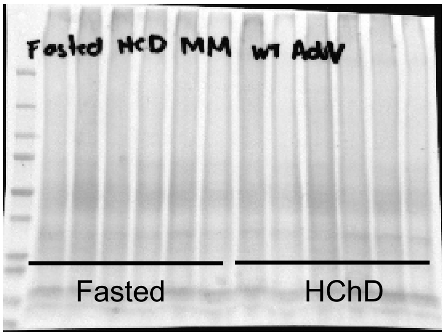

Chemiluminescence

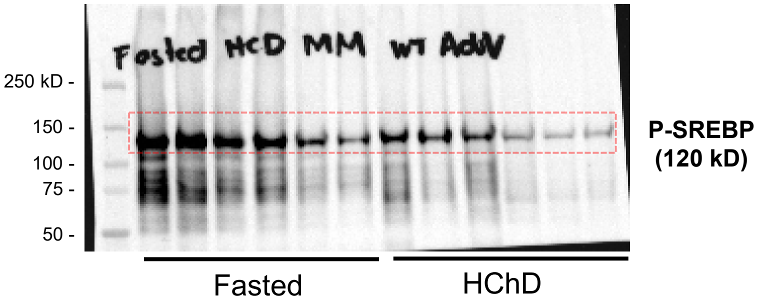

Ponceau S Nuclear Extract (NEX)

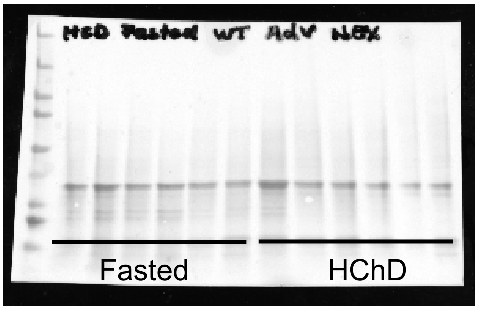

Chemiluminescence

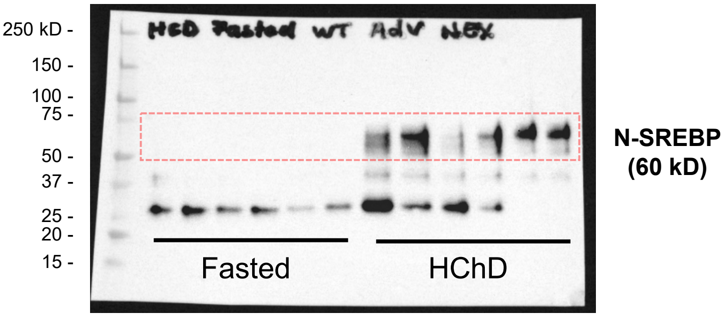

Supplementary Figure 14. Uncropped and unedited Western Blot images Figure 4B.

Figure 4E. Insulin signaling labeled western blot images Fasted vs. High Carbohydrate Diet

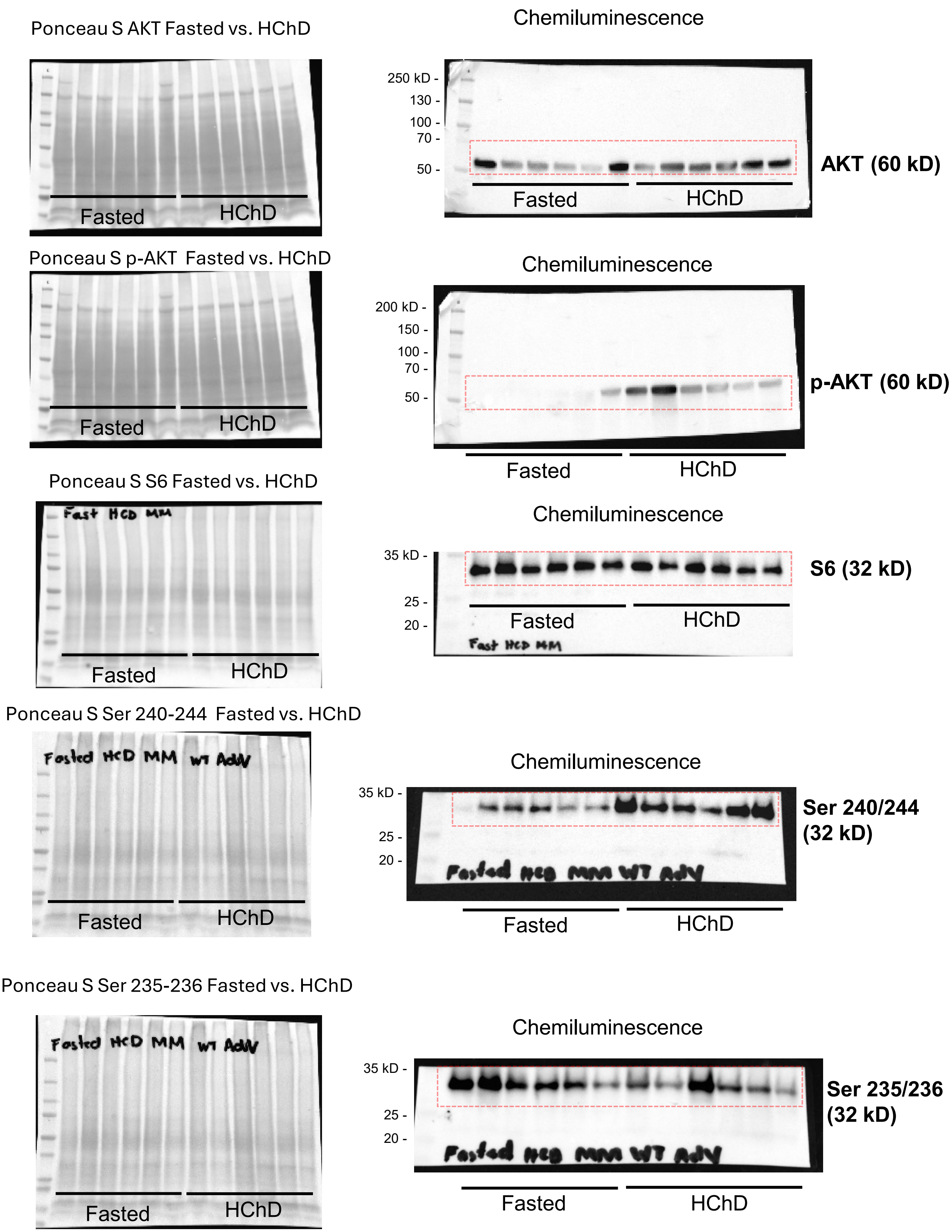

Supplementary Figure 14. Uncropped and unedited Western Blot images Figure 4E.

Figure 4G. Flag-SREBP-1c labeled western blot images uFA diet vs. sFA diet

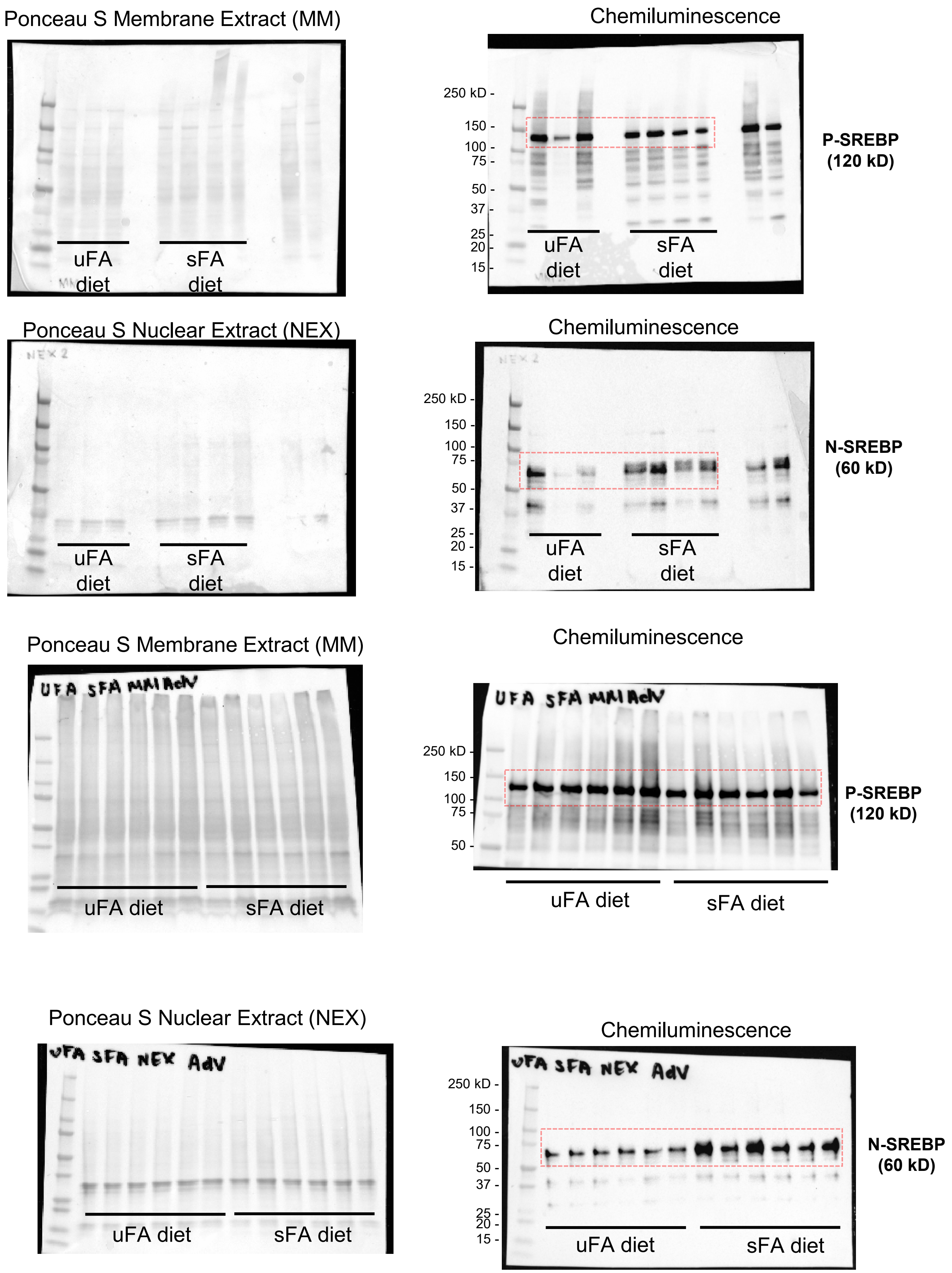

Supplementary Figure 14. Uncropped and unedited Western Blot images Figure 4G.

Figure 4J. Insulin signaling labeled western blot images uFA vs sFA diet

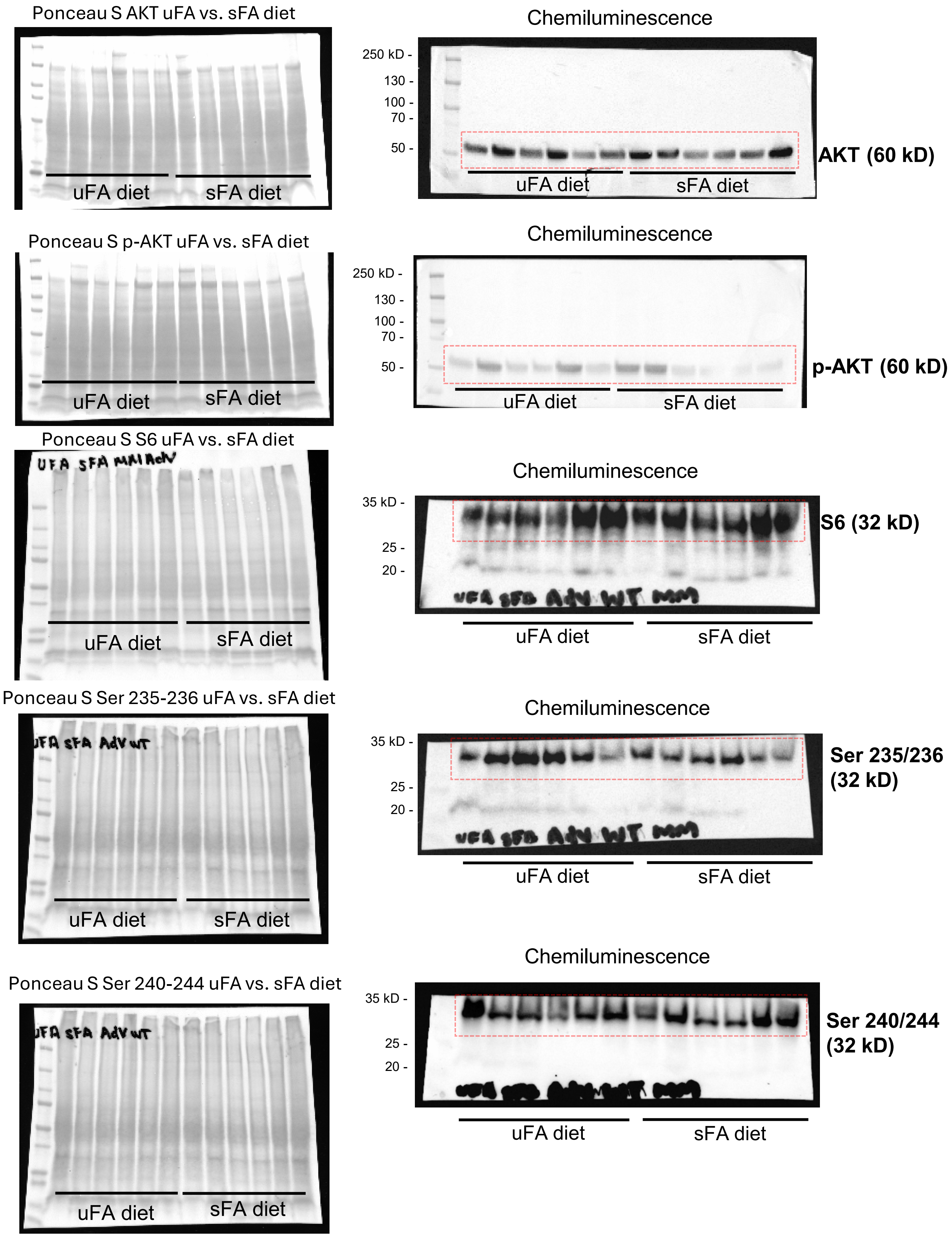

Supplementary Figure 14. Uncropped and unedited Western Blot images Figure 4J.

Figure 5.

Figure 5B. Flag-SREBP-1c labeled western blot images

Ponceau P-SREBP-1c

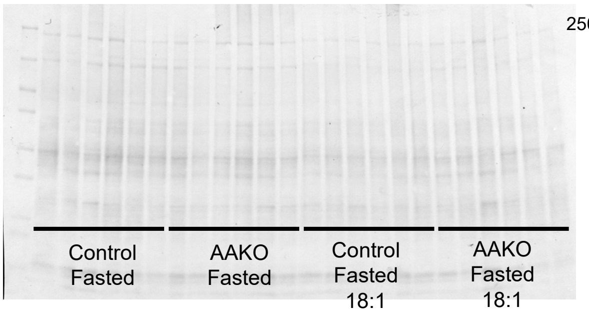

P-SREBP-1c
Chemiluminescence

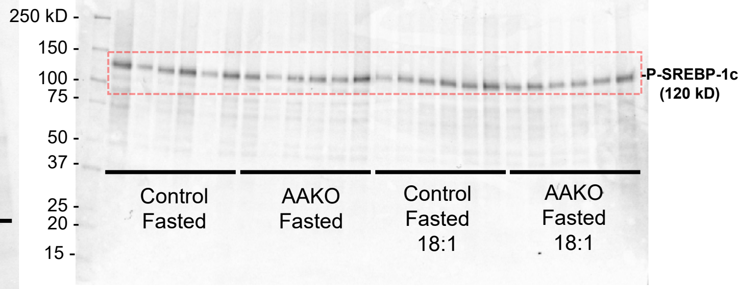

Ponceau N-SREBP-1c

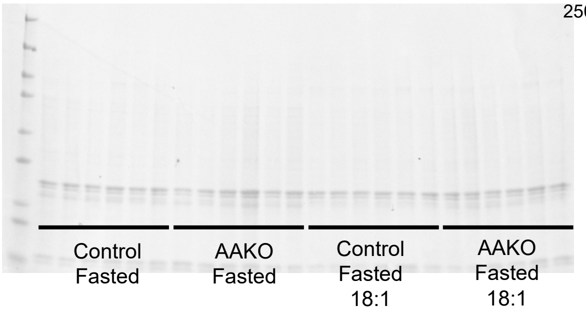

N-SREBP-1c
Chemiluminescence

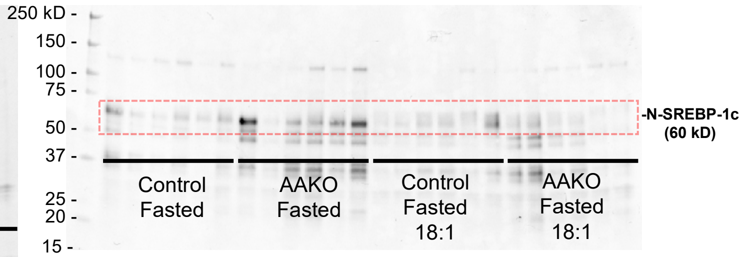

Supplementary Figure 14. Uncropped and unedited Western Blot images Figure 5B.

Figure 5D. Insulin signaling labeled western blot images

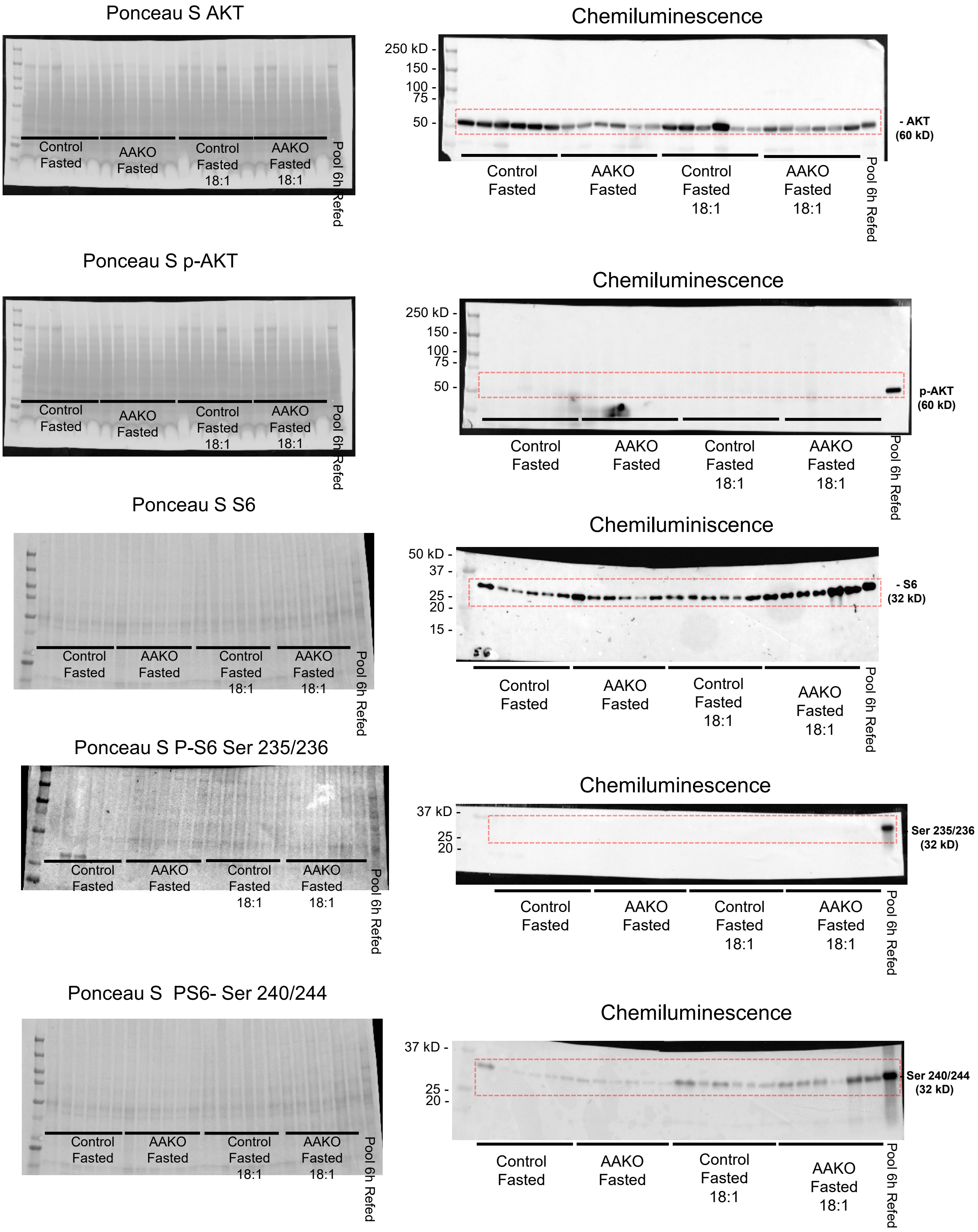

Supplementary Figure 14. Uncropped and unedited Western Blot images Figure 5D.
