# Supplementary material for: Lipolysis-derived fatty acids are needed for homeostatic control of sterol element-binding protein-1c driven hepatic lipogenesis
Source: Commun Biol. 2025 Apr 9;8:588. doi: 10.1038/s42003-025-08002-1 (PMC11982389; doi:10.1038/s42003-025-08002-1)
Supplement: Supplementary file 4 — Description of Additional Supplementary Materials [file 42003_2025_8002_MOESM4_ESM.pdf]

Description of Additional Supplementary Files

File name: Supplementary Data 1

Description: Source data underlying the graphs in the main figures

File name: Supplementary Data 2

Description: Source data underlying the graphs in the supplementary figures
