# Supplementary material for: Lipolysis-derived fatty acids are needed for homeostatic control of sterol element-binding protein-1c driven hepatic lipogenesis
Source: Commun Biol. 2025 Apr 9;8:588. doi: 10.1038/s42003-025-08002-1 (PMC11982389; doi:10.1038/s42003-025-08002-1)
Supplement: Supplementary file 5 — Reporting summary [file 42003_2025_8002_MOESM5_ESM.pdf]

nature portfolio

Corresponding author(s): Paul Vesely

Last updated by author(s): 2025.03.15

Reporting Summary

Nature Portfolio wishes to improve the reproducibility of the work that we publish. This form provides structure for consistency and transparency in reporting. For further information on Nature Portfolio policies, see our Editorial Policies and the Editorial Policy Checklist.

Statistics

For all statistical analyses, confirm that the following items are present in the figure legend, table legend, main text, or Methods section.

n/a	Confirmed	
☐	☒	The exact sample size (*n*) for each experimental group/condition, given as a discrete number and unit of measurement
☐	☒	A statement on whether measurements were taken from distinct samples or whether the same sample was measured repeatedly
☐	☒	The statistical test(s) used AND whether they are one- or two-sided<br>*Only common tests should be described solely by name; describe more complex techniques in the Methods section.*
☒	☐	A description of all covariates tested
☒	☐	A description of any assumptions or corrections, such as tests of normality and adjustment for multiple comparisons
☐	☒	A full description of the statistical parameters including central tendency (e.g. means) or other basic estimates (e.g. regression coefficient) AND variation (e.g. standard deviation) or associated estimates of uncertainty (e.g. confidence intervals)
☐	☒	For null hypothesis testing, the test statistic (e.g. *F*, *t*, *r*) with confidence intervals, effect sizes, degrees of freedom and *P* value noted<br>*Give P values as exact values whenever suitable.*
☒	☐	For Bayesian analysis, information on the choice of priors and Markov chain Monte Carlo settings
☒	☐	For hierarchical and complex designs, identification of the appropriate level for tests and full reporting of outcomes
☐	☒	Estimates of effect sizes (e.g. Cohen's *d*, Pearson's *r*), indicating how they were calculated

Our web collection on statistics for biologists contains articles on many of the points above.

Software and code

Policy information about availability of computer code

Data collection	Microsoft Excell was used for data collection
Data analysis	Statistical analysis between 2 groups of biological replicates were performed using Student’s 2-tailed t test, using GraphPad Prism, version 8.2.0. Outlier analysis was performed using Grubb’s test in GraphPad (https://www.graphpad.com/quickcalcs/grubbs1/), with α = 0.05. Linear correlations were determined by Pearson’s correlation coefficient. (Holm Sidak method, alpha 0.05).

For manuscripts utilizing custom algorithms or software that are central to the research but not yet described in published literature, software must be made available to editors and reviewers. We strongly encourage code deposition in a community repository (e.g. GitHub). See the Nature Portfolio guidelines for submitting code & software for further information.

Data

Policy information about availability of data

All manuscripts must include a data availability statement. This statement should provide the following information, where applicable:

- Accession codes, unique identifiers, or web links for publicly available datasets
- A description of any restrictions on data availability
- For clinical datasets or third party data, please ensure that the statement adheres to our policy

Data availability statement included in the manuscript.

Human research participants

Policy information about studies involving human research participants and Sex and Gender in Research.

Reporting on sex and gender	NA
Population characteristics	NA
Recruitment	NA
Ethics oversight	NA

Note that full information on the approval of the study protocol must also be provided in the manuscript.

Field-specific reporting

Please select the one below that is the best fit for your research. If you are not sure, read the appropriate sections before making your selection.

☒ Life sciences ☐ Behavioural & social sciences ☐ Ecological, evolutionary & environmental sciences

For a reference copy of the document with all sections, see nature.com/documents/nr-reporting-summary-flat.pdf

Life sciences study design

All studies must disclose on these points even when the disclosure is negative.

Sample size	All sample sizes are described within the manuscript. Where applicable, dot plots detailing each biological replicate are shown. Power calculations were performed to assess sample sizes..
Data exclusions	Outlier analzsis was performed using Grubbss Test. Outliers are shown within the respective graphs as grey dots in the dot plots, however, not included in the statistics.
Replication	All attempts at replication were successful.
Randomization	After considering animals of suitable age and balenced ratio of sexes, experimental and control groups were allocated randomly.
Blinding	The investigators were blinded to group allocation during experiments in that we identified mice by ear tag numbering and not by group allocation.

Behavioural & social sciences study design

All studies must disclose on these points even when the disclosure is negative.

Study description	NA
Research sample	NA
Sampling strategy	NA
Data collection	NA
Timing	NA
Data exclusions	NA
Non-participation	NA
Randomization	NA

Ecological, evolutionary & environmental sciences study design

All studies must disclose on these points even when the disclosure is negative.

Study description	NA
Research sample	NA
Sampling strategy	NA
Data collection	NA
Timing and spatial scale	NA
Data exclusions	NA
Reproducibility	NA
Randomization	NA
Blinding	NA

Did the study involve field work? ☐ Yes ☒ No

Field work, collection and transport

Field conditions	NA
Location	NA
Access & import/export	NA
Disturbance	NA

Reporting for specific materials, systems and methods

We require information from authors about some types of materials, experimental systems and methods used in many studies. Here, indicate whether each material, system or method listed is relevant to your study. If you are not sure if a list item applies to your research, read the appropriate section before selecting a response.

Materials & experimental systems

n/a	Involved in the study	
☐	☒	Antibodies
☐	☒	Eukaryotic cell lines
☒	☐	Palaeontology and archaeology
☐	☒	Animals and other organisms
☒	☐	Clinical data
☒	☐	Dual use research of concern

Methods

n/a	Involved in the study	
☒	☐	ChIP-seq
☒	☐	Flow cytometry
☒	☐	MRI-based neuroimaging

Antibodies

Antibodies used

Anti-SREBP-1c, clone 2A4. (Mouse monoclonal) Abcam (ab3259)
Anti-SREBP-1c clone 20B12. (Rabbit monoclonal) Merck (MABS1987)
Anti-FLAG® M2 (Mouse monoclonal) Sigma-Aldrich (F3165)
Anti-FLAG® M2-Peroxidase (HRP) (Mouse monoclonal) Sigma-Aldrich (A8592)
Anti-GFP- avictoria antibody. (Rabbit polyclonal) Abcam (ab290)-
Anti-GM130, clone 35. (Mouse monoclonal) BD Biosiences (610822)
Anti-Rabbit IgG (H+L), Alexa Fluor 488 coupled (Goat polyclonal) Invitrogen A11034
Anti-Mouse IgG (H+L), Alexa Fluor 594 coupled. (Mouse polyclonal) Invitrogen A11005

	Anti-mouse Immunoglobulins/HRP. (Goat polyclonal) Dako (P0477)<br>Anti-rabbit Immunoglobulins/HRP (Pig polyclonal) Dako (P0217)<br>Anti-SREBP-1c, clone 2A4. (Mouse monoclonal) Abcam (ab3259)<br>Anti-SREBP-1c clone 20B12. (Rabbit monoclonal) Merck (MABS1987)<br>Anti-FLAG® M2 (Mouse monoclonal) Sigma-Aldrich (F3165)<br>Anti-FLAG® M2-Peroxidase (HRP) (Mouse monoclonal) Sigma-Aldrich (A8592)<br>Anti-GFP- avictoria antibody. (Rabbit polyclonal) Abcam (ab290)-<br>Anti-GM130, clone 35. (Mouse monoclonal) BD Biosiences (610822)<br>Anti-Rabbit IgG (H+L), Alexa Fluor 488 coupled (Goat polyclonal) Invitrogen A11034<br>Anti-Mouse IgG (H+L), Alexa Fluor 594 coupled. (Mouse polyclonal) Invitrogen A11005<br>Anti-mouse Immunoglobulins/HRP. (Goat polyclonal) Dako (P0477)<br>Anti-rabbit Immunoglobulins/HRP (Pig polyclonal) Dako (P0217)
Validation	Anti-SREBP-1c, clone 2A4. (Mouse monoclonal) Abcam (ab3259)- Validated in WB and tested in Human samples by Abcam.<br>Anti-SREBP-1c clone 20B12. (Rabbit monoclonal) Merck (MABS1987)-Evaluated by Western Blotting in HepG2 cell lysates by Merck.<br>Anti-FLAG® M2 (Mouse monoclonal) Sigma-Aldrich (F3165) - Western blot from an E. coli crude cell lysate by Sigma-Aldrich.<br>Anti-GFP- antibody. (Rabbit polyclonal) Abcam (ab290)- Validated in ICC, IP, EM, ELISA, WB, IHC-FoFr, IHC-P, IHC-Fr, IHC-FrFl and tested in GFP-tagged samples by Abcam.<br>Anti-GM130, clone 35. (Mouse monoclonal) BD Biosiences (610822)-Validation not provided by BD Biosciences. Validated in study: Shao et al., J Lipid Res 2016.<br>Anti-Rabbit IgG (H+L), Alexa Fluor 488 coupled (Goat polyclonal) Invitrogen A11034- Validation not provided<br>Anti-Mouse IgG (H+L), Alexa Fluor 594 coupled. (Mouse polyclonal) Invitrogen A11005- Validation not ptovided<br>Anti-rabbit Immunoglobulins/HRP (Pig polyclonal) Dako (P0217) - Validation not provided

Eukaryotic cell lines

Policy information about cell lines and Sex and Gender in Research

Cell line source(s)	Cell line U2OS ATCC (HTB96), HepG2 (HB-8065)
Authentication	Autenticated by American Type Culture Collection
Mycoplasma contamination	The cell lines were tested for mycoplasma by qPCR on a regular basis.
Commonly misidentified lines<br>(See ICLAC register)	NA

Palaeontology and Archaeology

Specimen provenance	NA
Specimen deposition	NA
Dating methods	NA

☐ Tick this box to confirm that the raw and calibrated dates are available in the paper or in Supplementary Information.

Ethics oversight	NA

Note that full information on the approval of the study protocol must also be provided in the manuscript.

Animals and other research organisms

Policy information about studies involving animals; ARRIVE guidelines recommended for reporting animal research, and Sex and Gender in Research

Laboratory animals	Mice were routinely fed ad libitum with a standard chow diet [(4.5% fat, 34% starch, 5.0% sugar and 22.0% protein) M-Z extrudate, V1126, Ssniff Spezialdiäten, Germany]. Mice of “fasted” groups were fasted for 12 h, from 7 p.m. to 7 a.m. Mice in “refed” groups were fasted for 12 h from 7 p.m. to 7 a.m. and then refed a high carbohydrate/low-fat diet (HChD, equivalent to TD 88122; Harlan Teklad, USA) up to 9 h. Mice fed FA enriched diets were fed ad libitum for 3 consecutive days with an unsaturated FA diet (100 g of chow powder food, 50 g of casein and 60 mL of flaxseed oil) or with a saturated FA diet (100 g of chow powder food, 50 g of casein and 60 mL of palm oil) and subsequently sacrificed. Mice injected with FA were fasted for 12 h from 7 p.m. to 7 a.m., or fasted and intravenously injected with bovine serum albumin complexed oleic acid (18:1, BSA-Oleate Monounsaturated FA Complex (5 mM) Item No. 29557, Cayman Chemical) or BSA-Palmitate Saturated FA Complex (5 mM) (Item No. 29558, Cayman Chemical) 3 and 9 h after food withdrawal. Mice were sacrificed 3 h after the second FA infusion.<br>Mouse strains used: WT: C57Bl/6J (own breeding, originally from Jackson lab). Genetically modified strains on C57Bl/6J background: AAKO, Adipose-tissue specific Atgl-knockout (Atglflox/flox, Adipoq-Cre) (Schoiswohl et al., 2015); ALKO, Liver specific Atgl-knockout (Atglflox/flox, Alb-Cre) (Wu et al., 2011).

Wild animals	NA
Reporting on sex	Findings apply to both sexes, groups were equally distributed by age and sex.
Field-collected samples	NA
Ethics oversight	All animal studies were performed in accordance with the guidelines and provisions of the Commission for Animal Experiments of the Austrian Ministry of Education, Science and Research (BMBWF). Approved animal applications and amendments include, BMBWF-66.007/0015-V/3b/2018; BMBWF-66.007/0004-V/3b/2019 and BMBWF-328 2020-0380.481.

Note that full information on the approval of the study protocol must also be provided in the manuscript.

Clinical data

Policy information about clinical studies
All manuscripts should comply with the ICMJE guidelines for publication of clinical research and a completed CONSORT checklist must be included with all submissions.

Clinical trial registration	NA
Study protocol	NA
Data collection	NA
Outcomes	NA

Dual use research of concern

Policy information about dual use research of concern

Hazards

Could the accidental, deliberate or reckless misuse of agents or technologies generated in the work, or the application of information presented in the manuscript, pose a threat to:

No	Yes	
☒	☐	Public health
☒	☐	National security
☒	☐	Crops and/or livestock
☒	☐	Ecosystems
☒	☐	Any other significant area

Experiments of concern

Does the work involve any of these experiments of concern:

No	Yes	
☒	☐	Demonstrate how to render a vaccine ineffective
☒	☐	Confer resistance to therapeutically useful antibiotics or antiviral agents
☒	☐	Enhance the virulence of a pathogen or render a nonpathogen virulent
☒	☐	Increase transmissibility of a pathogen
☒	☐	Alter the host range of a pathogen
☒	☐	Enable evasion of diagnostic/detection modalities
☒	☐	Enable the weaponization of a biological agent or toxin
☒	☐	Any other potentially harmful combination of experiments and agents

ChIP-seq

Data deposition

☐ Confirm that both raw and final processed data have been deposited in a public database such as GEO.

☐ Confirm that you have deposited or provided access to graph files (e.g. BED files) for the called peaks.

Data access links<br>*May remain private before publication.*	NA

Files in database submission	NA
Genome browser session (e.g. UCSC)	NA

Methodology

Replicates	NA
Sequencing depth	NA
Antibodies	NA
Peak calling parameters	NA
Data quality	NA
Software	NA

Flow Cytometry

Plots

Confirm that:

- [ ] The axis labels state the marker and fluorochrome used (e.g. CD4-FITC).
- [ ] The axis scales are clearly visible. Include numbers along axes only for bottom left plot of group (a 'group' is an analysis of identical markers).
- [ ] All plots are contour plots with outliers or pseudocolor plots.
- [ ] A numerical value for number of cells or percentage (with statistics) is provided.

Methodology

Sample preparation	NA
Instrument	NA
Software	NA
Cell population abundance	NA
Gating strategy	NA

- [ ] Tick this box to confirm that a figure exemplifying the gating strategy is provided in the Supplementary Information.

Magnetic resonance imaging

Experimental design

Design type	NA
Design specifications	NA
Behavioral performance measures	NA

Acquisition

Imaging type(s)	NA
Field strength	NA
Sequence & imaging parameters	NA
Area of acquisition	NA

Diffusion MRI ☐ Used ☐ Not used

Preprocessing

Preprocessing software	NA
Normalization	NA
Normalization template	NA
Noise and artifact removal	NA
Volume censoring	NA

Statistical modeling & inference

Model type and settings	NA
Effect(s) tested	NA

Specify type of analysis: ☐ Whole brain ☐ ROI-based ☐ Both

Statistic type for inference (See Eklund et al. 2016)	NA
Correction	NA

Models & analysis

n/a	Involved in the study
☒	☐ Functional and/or effective connectivity
☒	☐ Graph analysis
☒	☐ Multivariate modeling or predictive analysis
